# Peripheral blood mononuclear cell phenotype and function are maintained after overnight shipping of whole blood

**DOI:** 10.1038/s41598-022-24550-6

**Published:** 2022-11-19

**Authors:** Renee K. Johnson, Brittany L. Overlee, Jessica A. Sagen, Charles L. Howe

**Affiliations:** 1 Translational Neuroimmunology Lab, Mayo Clinic, Guggenheim 1542C, 200 First St SW, Rochester, MN 55905 USA; 2 Department of Neurology, Mayo Clinic, Rochester, MN 55905 USA; 3 Center for MS and Autoimmune Neurology, Mayo Clinic, Rochester, MN 55905 USA; 4 Division of Experimental Neurology, Mayo Clinic, Rochester, MN 55905 USA

**Keywords:** Biomarkers, Translational research, Immunological techniques

## Abstract

Same day processing of biospecimens such as blood is not always feasible, which presents a challenge for research programs seeking to study a broad population or to characterize patients with rare diseases. Recruiting sites may not be equipped to process blood samples and variability in timing and technique employed to isolate peripheral blood mononuclear cells (PBMCs) at local sites may compromise reproducibility across patients. One solution is to send whole blood collected by routine phlebotomy via overnight courier to the testing site under ambient conditions. Determining the impact of shipping on subsequent leukocyte responses is a necessary prerequisite to any experimental analysis derived from transported samples. To this end, whole blood was collected from healthy control subjects and processed fresh or at 6, 24 and 48 h after collection and handling under modeled shipping conditions. At endpoint, whole blood was assessed via a complete blood count with differential and immunophenotyped using a standardized panel of antibodies [HLADR, CD66b, CD3, CD14, CD16]. PBMCs and neutrophils were isolated from whole blood and subjected to ex vivo stimulation with lipopolysaccharide and heat-killed Staphylococcus aureus. Stimulated release of cytokines and chemokines was assessed by cytometric bead array. RNA was also isolated from PBMCs to analyze transcriptional changes induced by shipping. The complete blood count with differential revealed that most parameters were maintained in shipped blood held for 24 h at ambient temperature. Immunophenotyping indicated preservation of cellular profiles at 24 h, although with broadening of some populations and a decrease in CD16 intensity on classical monocytes. At the transcriptional level, RNAseq analysis identified upregulation of a transcription factor module associated with inflammation in unstimulated PBMCs derived from whole blood shipped overnight. However, these changes were limited in both scale and number of impacted genes. Ex vivo stimulation of PBMCs further revealed preservation of functional responses in cells isolated from shipped blood held for 24 h at ambient temperature. However, neutrophil responses were largely abrogated by this time. By 48 h neither cell population responded within normal parameters. These findings indicate that robust immunophenotyping and PBMC stimulated response profiles are maintained in whole blood shipped overnight and processed within 24 h of collection, yielding results that are representative of those obtained from the sample immediately following venipuncture. This methodology is feasible for many patient recruitment sites to implement and allows for sophisticated immunological analysis of patient populations derived from large geographic areas. With regard to rare disease research, this meets a universal need to enroll patients in sufficient numbers for immunoprofiling and discovery of underlying pathogenic mechanisms.

## Introduction

Cell-based functional immunological assays provide invaluable information both as diagnostic and prognostic biomarkers and as indicators of therapeutic efficacy in clinical trials. However, such assays require preparation and manipulation of viable leukocytes that are not activated or suppressed by the methods of collection, processing, or storage. Previous studies have suggested that a delay between time of collection and the preparation and cryopreservation of lymphocytes results in impaired function and decreased recovery^1^. Likewise, storing whole blood at 4 °C has been shown to reduce leukocyte viability, function, and recovery following cryopreservation^2–6^, although other studies have observed relative preservation of phenotype^7^. On the other hand, storage at room temperature has been reported to have no effect on multiparametric immunophenotyping^8^ or viability^9^ but may alter subset representation^2^. Based on the need to perform ex vivo stimulation of isolated peripheral blood mononuclear cells (PBMCs) from patients at geographically disparate sites, we sought to clarify the effect of overnight shipping at ambient temperature on immunophenotype, transcriptional profile, and stimulated inflammatory cytokine and chemokine production, with an emphasis on monocytic responses.

## Results

### Cell counts and immunophenotype are preserved after overnight shipping

Complete blood count with differential^10^ using fresh blood (0 h) and blood held at ambient temperature for 6, 24, or 48 h revealed that most parameters were maintained throughout the timecourse (Table 1) (F_(3,420)_ = 0.580, *P* = 0.628 by two-way ANOVA for time). Indeed, time accounted for less than 0.5% of the total variance observed in the data. Total white blood cell counts decreased by ~ 6% at 24 h and individual counts of lymphocytes, monocytes, neutrophils, eosinophils, and basophils reflected this small decrease. Other parameters such as red blood cell counts, hemoglobin levels, and platelet counts were also preserved at 24 h. The only significant changes were observed in monocytes at 48 h and in basophils at 6 and 24 h. However, the small number of these cells leads to an exaggerated variance and it is reasonable to conclude that overnight shipping does not compromise the overall cellular differential.

**Table 1:** Complete blood count and differential.

	0 h	6 h	24 h	48 h	Normal Range	
Total WBC	6.2 ± 0.8	6.2 ± 0.8, *P* > 0.999	5.8 ± 0.7, *P* = 0.999	5.5 ± 0.7, *P* = 0.999	3.4–9.6	× 10^3^/μL
Lymphocytes	2.0 ± 0.3	2.0 ± 0.3, *P* > 0.999	1.8 ± 0.3, *P* > 0.999	1.7 ± 0.3, *P* > 0.999	1.0–3.1	× 10^3^/μL
Monocytes	0.5 ± 0.2	0.4 ± 0.1, *P* = 0.915	0.4 ± 0.1, *P* = 0.326	0.2 ± 0.0, *P* = 0.003	0.3–0.8	× 10^3^/μL
Neutrophils	3.4 ± 0.4	3.5 ± 0.5, *P* = 0.999	3.3 ± 0.4, *P* = 0.991	3.2 ± 0.4, *P* = 0.949	1.5–6.5	× 10^3^/μL
Eosinophils	0.3 ± 0.1	0.2 ± 0.1, *P* > 0.999	0.3 ± 0.1, *P* > 0.999	0.3 ± 0.1, *P* > 0.999	0.03–0.5	× 10^3^/μL
Basophils	0.02 ± 0.01	0.03 ± 0.01, *P* > 0.999	0.03 ± 0.01, *P* > 0.999	0.02 ± 0.01, *P* > 0.999	0.01–0.08	× 10^3^/μL
RBC	5.0 ± 0.3	5.1 ± 0.4, *P* > 0.999	5.1 ± 0.4, *P* > 0.999	5.2 ± 0.4, *P* > 0.999	3.9–5.7	× 10^6^/μL
Hemoglobin	13.8 ± 0.7	13.8 ± 0.7, *P* > 0.999	13.9 ± 0.6, *P* > 0.999	13.9 ± 0.7, *P* > 0.999	11.6–16.6	g/dL
Hematocrit	42.5 ± 1.6	43.5 ± 1.7, *P* = 0.997	44.6 ± 2.1, *P* = 0.973	46.4 ± 2.0, *P* = 0.859	35.5–48.6	%
MCV	85.4 ± 5.6	85.8 ± 5.5, *P* = 0.999	87.9 ± 5.6, *P* = 0.955	90.1 ± 5.8, *P* = 0.779	78–98	fL
MCH	27.7 ± 2.2	27.3 ± 2.3, *P* = 0.999	27.4 ± 2.3, *P* > 0.999	27.1 ± 2.0, *P* = 0.999	27–31	pg
MCHC	32.4 ± 0.8	31.7 ± 1.1, *P* = 0.999	31.1 ± 0.7, *P* = 0.993	30.0 ± 0.5, *P* = 0.960	32–36	g/dL
RDW-SD	40.0 ± 0.9	40.1 ± 1.0, *P* > 0.999	43.4 ± 1.3, *P* = 0.895	47.3 ± 1.6, *P* = 0.483	40–55	fL
Platelets	259 ± 28	275 ± 36, *P* = 0.022	272 ± 39, *P* = 0.094	285 ± 22, *P* < 0.001	135–371	× 10^3^/μL
MPV	9.2 ± 0.7	9.8 ± 0.7, *P* = 0.999	10.2 ± 0.8, *P* = 0.997	10.9 ± 0.8, *P* = 0.984	7–12	fL

Subjects were 8 healthy adults of both biological sexes between the ages of 25 and 52. Values are shown as the mean ± 95% confidence interval. Significance is Dunnett’s pairwise comparison to the 0 h values. WBC = white blood cells; RBC = red blood cells; MCV = mean corpuscular volume; MCH = mean corpuscular hemoglobin; MCHC = mean corpuscular hemoglobin concentration; RDW-SD = RBC distribution width size; MPV = mean platelet volume. Normal ranges are those established by the Mayo Clinic Laboratories.

**Figure 1:**
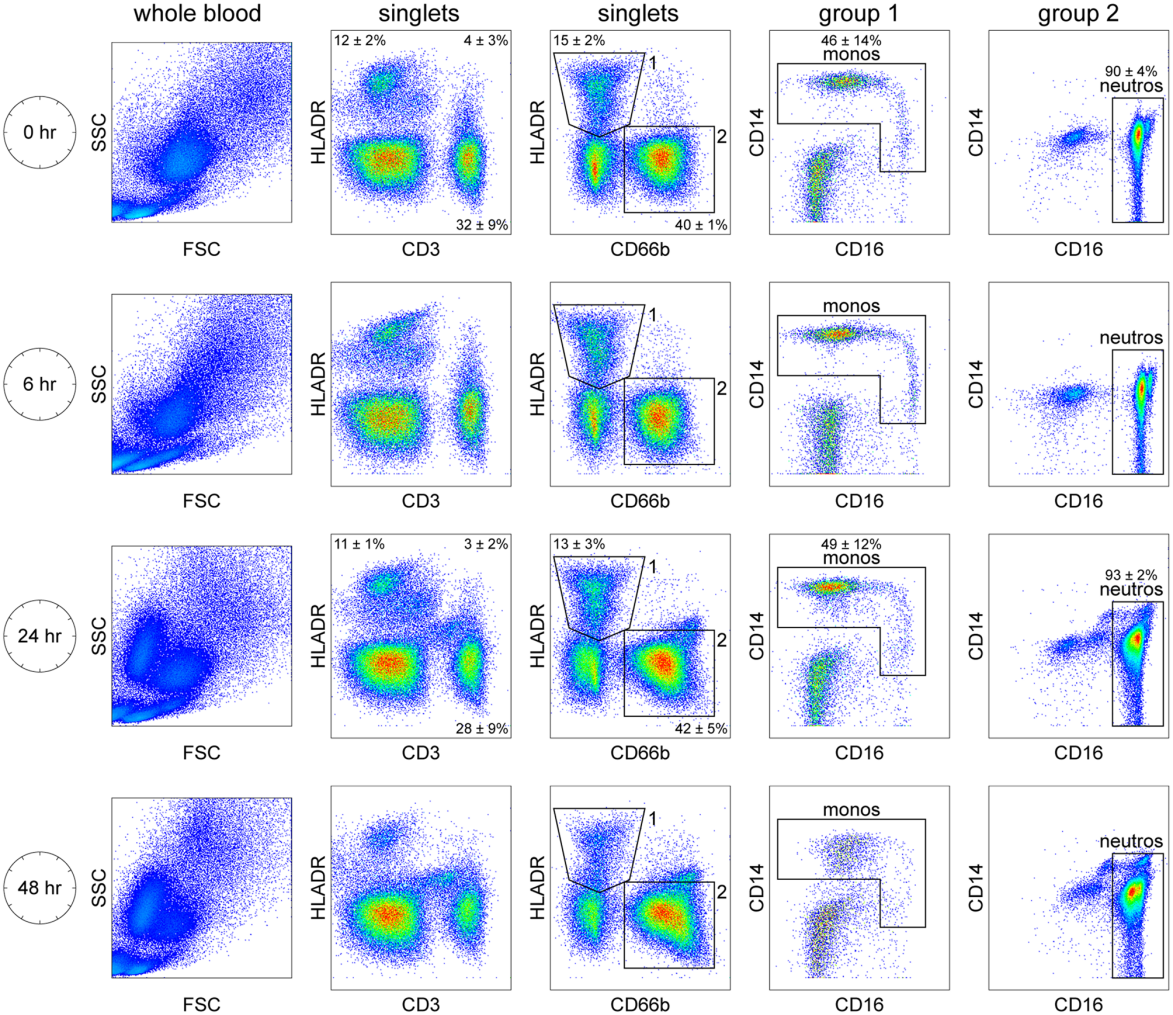
Leukocyte counts and immunophenotype are maintained after overnight (24 h) shipping. Whole blood collected in EDTA tubes was either processed immediately after venipuncture (0 h) or was held for 6, 24, or 48 h at ambient temperature under conditions that modeled courier shipping. At endpoint whole blood was labeled with antibodies against HLADR, CD3, CD66b, CD14, and CD16, followed by red blood cell lysis and brief fixation. Flow cytometric analysis of scatter profile revealed that by 24 h the granulocyte population (FSC^hi^SSC^hi^) had split into two clusters. Singlets were identified based on FSC area x FSC height and all subsequent analyses were performed on total singlets without further gating. The overall pattern and density of HLADR^+^ or CD3^+^ cells was maintained through 24 h. Likewise, HLADR^+^CD66b^-^ (group 1) and HLADR^-^CD66b^+^ (group 2) populations were largely maintained. HLADR^+^CD66b^-^ cells were further gated on CD14 and CD16 to reveal monocytes (monos), broadly defined as CD14^+^CD16^lo/neg^ classical monocytes, CD14^lo^CD16^+^ non-classical monocytes, and CD14^+/mid^CD16^+^ inflammatory monocytes. HLADR^-^CD66b^+^ cells were largely comprised of CD16^++^CD14^lo/neg^ neutrophils (neutros). Percents for relevant populations are shown at 0 h and 24 h derived from 4 subjects (mean ± 95%CI); 6 h and 48 h show representative plots.

Flow cytometric analysis of whole blood following red blood cell lysis with Versalyse indicated that by 24 h the granulocyte population had split into two groups, with one exhibiting normal forward and side scatter profiles and the other exhibiting decreased size and altered granularity (Fig. 1). Immunophenotyping of whole blood indicated that CD3^+^ lymphocytes and HLADR^+^ cells retained a largely normal profile, although with time there was a general broadening of the populations and by 48 h there was a substantial reduction in HLADR^+^ cells (Fig. 1). Further segregation revealed that HLADR^+^CD66b^-^ cells (group 1) containing CD14^+^ monocytes retained a largely normal CD14/CD16 expression profile through 24 h, though the overall intensity of CD16 on CD14^hi^CD16^neg/lo^ classical monocytes decreased and the populations of CD14^hi^CD16^+^ inflammatory monocytes and CD14^lo/mid^CD16^+^ nonclassical monocytes broadened^11^. Notably, given the monocyte decrease measured in the complete blood count, the number of HLADR^+^CD66b^-^CD14^+^ monocytes did not drop off at 6 or 24 h in the flow analysis. By 48 h monocytes were greatly reduced and did not display a normal immunophenotype. In addition, the HLADR^−^CD66b^+^ cells (group 2) showed an unexpected preservation of the CD14^neg/lo^CD16^++^ neutrophil phenotype at 24 and 48 h, despite the shift in scatter profile indicative of activation and/or degranulation. Quantitation of specific populations between 0 and 24 h verified preservation of phenotypes: HLADR^+^CD3^−^ *P* = 0.2873; HLADR^-^CD3^+^ P = 0.3818; HLADR^+^CD66b^-^ P = 0.4708; HLADR^-^CD66b^+^ *P* = 0.6166; HLADR^+^CD66b^-^CD14^+^ monocytes *P* = 0.1387; HLADR^-^CD66b^+^CD14^neg/lo^CD16^++^ neutrophils *P* = 0.1798 (n = 4 subjects; paired t-test).

### Peripheral blood mononuclear cells undergo changes in scatter profile and become contaminated by neutrophils after overnight shipping

Downstream immune assays, such as ex vivo stimulation, frequently require separation of mononuclear cells (such as lymphocytes, monocytes, dendritic cells) from polymorphonuclear cells (such as neutrophils, eosinophils). In broad terms, gradient centrifugation through a medium with a density of 1.077 g/mL cleanly separates mononuclear and polymorphonuclear cells^12,13^. Using this process with whole blood also yields acellular plasma. In parallel, purification of neutrophils, per se, is readily accomplished by immunomagnetic selection of whole blood. The plasma fraction, representing factors that were present in circulation at the time of collection plus any additional factors released by cells during the shipping period, showed only limited evidence of either large increases or decreases in all of the cytokines and chemokines analyzed (Table 2). In order to control for marked differences in concentrations between analytes, each measurement was recast as percent of the mean of the 0 h plasma value per analyte. Two-way ANOVA for all 10 inflammatory factors revealed a significant change through time (F_(3,160)_ = 10.50, *P* < 0.0001), though time (vs analyte) only accounted for 8% of the total variance. Dunnett’s pairwise comparisons through time for each individual factor (fresh vs 6 h, fresh vs 24 h, fresh vs 48 h) indicated that all changes were insignificant except for CXCL8 at 48 h (*P* = 0.0012) and IL1β at 24 h (*P* < 0.0001) and 48 h (*P* < 0.0001). Notably, in contrast to IL1β, IL6 and TNFα were essentially unchanged at all timepoints.

**Table 2:** Plasma cytokines and chemokines.

pg/mL	0 h	6 h	24 h	48 h
CCL2 (pg/mL)	10.5 ± 3.5	18.2 ± 7.9, *P* = 0.874	23.0 ± 4.2, *P* = 0.627	28.9 ± 7.1, *P* = 0.329
CCL5 (ng/mL)	2.0 ± 4.4	3.0 ± 3.8, *P* = 0.962	3.6 ± 3.6, *P* = 0.841	5.6 ± 2.8, *P* = 0.318
CXCL8 (pg/mL)	3.6 ± 3.6	4.0 ± 4.4, *P* = 0.999	6.5 ± 6.5, *P* = 0.8365	19.1 ± 25.1, *P* = 0.001
CXCL9 (pg/mL)	25.4 ± 22.4	28.3 ± 26.3, *P* = 0.999	30.5 ± 28.7, *P* = 0.997	30.8 ± 25.3, *P* = 0.996
CXCL10 (pg/mL)	96.8 ± 50.9	83.0 ± 49.8, *P* = 0.999	72.2 ± 40.3, *P* = 0.993	65.1 ± 35.6, *P* = 0.986
IL1β (pg/mL)	0.5 ± 0.8	0.2 ± 0.4, *P* = 0.910	3.5 ± 4.4, *P* < 0.0001	5.4 ± 2.9, *P* < 0.0001
IL6 (pg/mL)	3.0 ± 3.8	2.3 ± 3.0, *P* = 0.994	2.9 ± 4.1, *P* > 0.999	2.6 ± 3.7, *P* = 0.999
IL10 (pg/mL)	1.8 ± 1.4	1.6 ± 1.0, *P* = 0.999	1.4 ± 1.9, *P* = 0.996	1.6 ± 1.8, *P* = 0.999
IL12p70 (pg/mL)	2.3 ± 1.5	2.9 ± 3.0, *P* = 0.995	2.0 ± 1.2, *P* = 0.999	2.8 ± 1.9, *P* = 0.995
TNFα (pg/mL)	2.2 ± 1.5	1.6 ± 1.5, *P* = 0.991	1.7 ± 1.0, *P* = 0.996	1.7 ± 1.0, *P* = 0.996

Data from 5 healthy donors: one male age 37, 4 females age 25, 30, 34, 41. Values are shown as the mean ± 95% confidence interval. Statistical significance is vs 0 h measurement, determined by Dunnett’s pairwise comparison.

**Figure 2:**
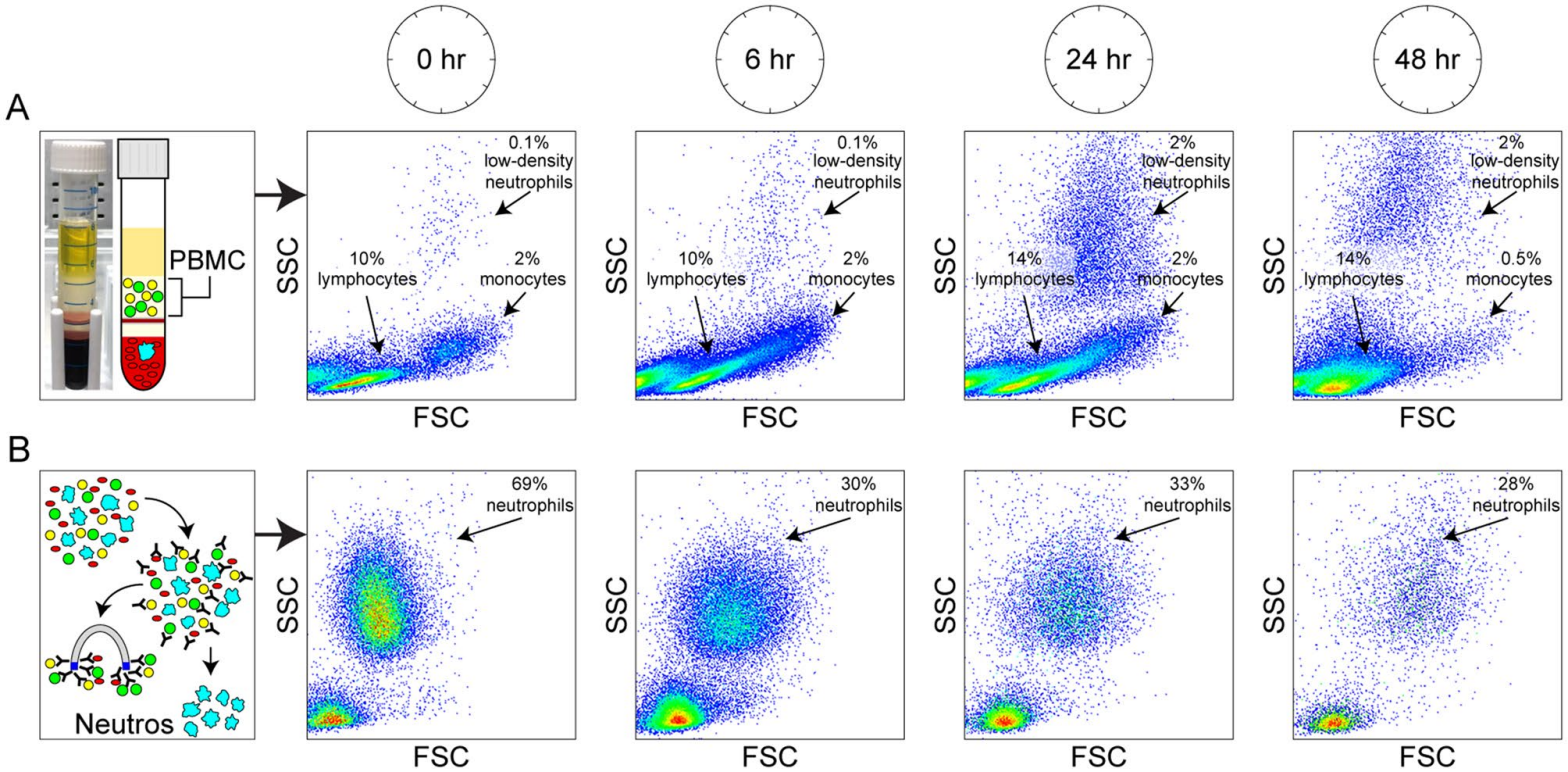
PBMCs change scatter profile and become contaminated by low-density neutrophils over time. (**A**) PBMCs were isolated from whole blood using a density gradient (top panel). The forward and side scatter profile of the isolated PBMCs was altered as early as 6 h after collection; this effect was maintained through 24 h and largely reflected a change in size and granularity in lymphocytes (central population moved rightward). By 48 h the scatter profile of the PBMCs had collapsed leftward. In parallel, by 24 h the PBMC fraction showed a large increase in FSC^h^iSSC^hi^ granulocytes, consistent with acquisition of a low density phenotype in neutrophils. (**B**) Neutrophils were enriched from whole blood using immunomagnetic negative selection (bottom panel). This population exhibited a marked dropout of FSC^hi^SSC^hi^ cells as early as 6 h. Percents for relevant populations are shown.

Gradient purification of mononuclear cells revealed a substantial change in scatter profile in this population as early as 6 h after collection (Fig. 2). This change persisted through 24 h and largely seems to represent a forward scatter increase in lymphocytes. By 48 h lymphocytes and monocytes exhibited considerable forward scatter decrease. However, despite the change in scatter profile, the relative percentage of lymphocytes and monocytes in the PBMC population was largely unchanged through 24 h. Of note, there was a progressive increase in neutrophils (high forward and side scatter) in the mononuclear cell fraction evident by 6 h and continuing through 24 and 48 h. This change is consistent with conversion of neutrophils to a low density state, either as the result of activation and degranulation or due to cellular swelling^14^. Immunomagnetic isolation of neutrophils also revealed a rapid change in cellular morphology at 6 h (increased forward scatter) and a considerable decrease in recovered neutrophils by this timepoint. Since the total number of HLADR^-^CD3^-^CD66b^+^CD14^lo^CD16^hi^ neutrophils in whole blood did not markedly change over time (Fig. 1) and given that negative selection based on removal of cells expressing non-granulocytic markers was used to isolate the cells, the reduction in neutrophils observed in Fig. 2 likely arises from a change in immunophenotype (for example, increased CD14 expression) or an increase in non-specific “stickiness” due to activation or death.

### Peripheral blood mononuclear cells upregulate inflammatory, stress, and cell death pathways after overnight shipping

**Figure 3:**
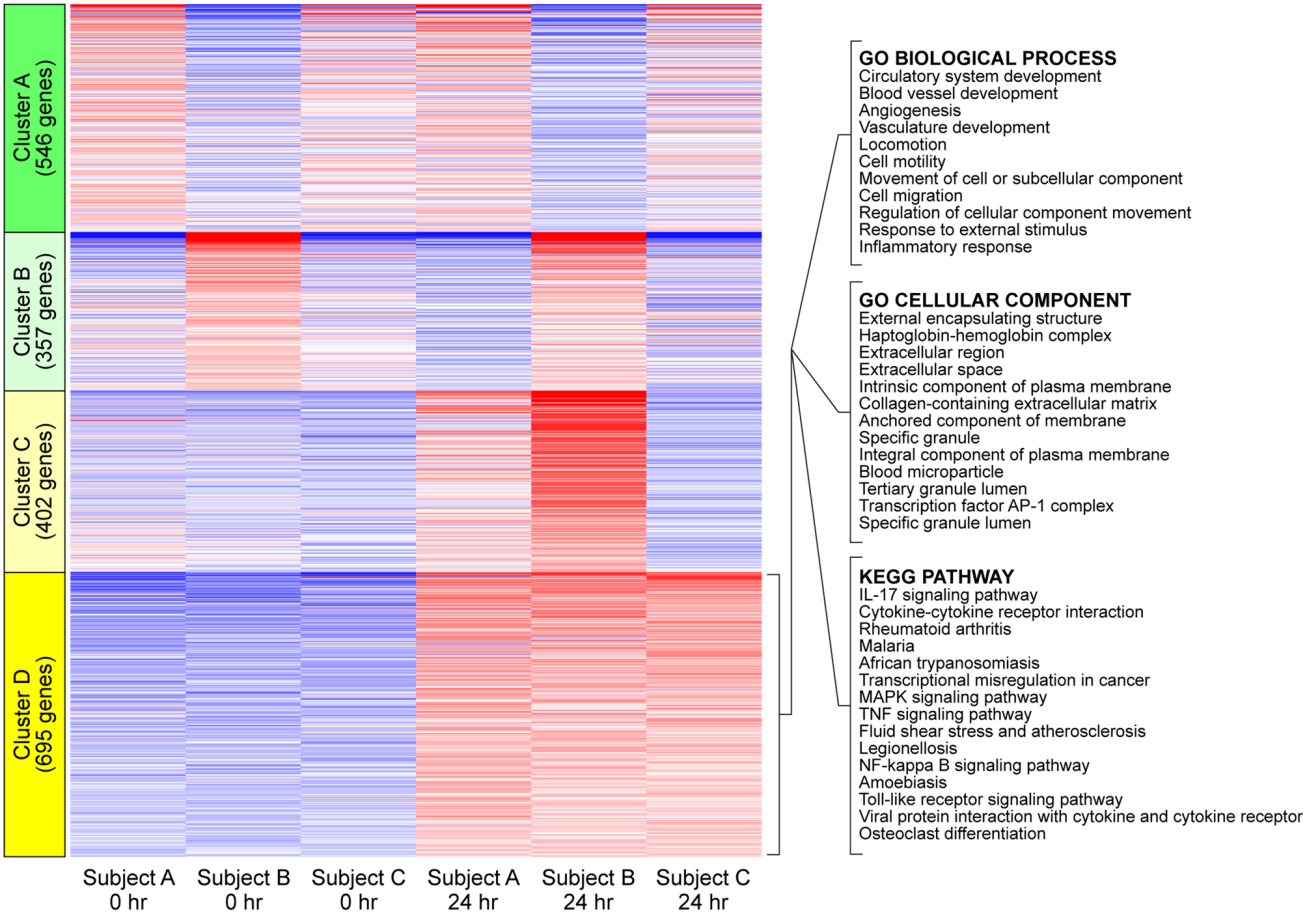
Exposure to overnight shipping conditions induces a transcriptional response in PBMCs. PBMCs were isolated from whole blood from 3 healthy controls at 0 or 24 h. RNAseq was used to characterize transcriptional changes. K-means clustering revealed 4 clusters in the 2000 most variable genes. Of these, only cluster D exhibited a uniform pattern of change at 24 h relative to 0 h in all 3 subjects, consistent with a transcriptional response associated with the handling conditions rather than inter-individual differences. Pathway analysis of the 695 genes in this cluster identified processes associated with signal transduction and inflammation. Transcriptional changes are heatmapped from blue (strongly downregulated) to red (highly upregulated).

**Figure 4:**
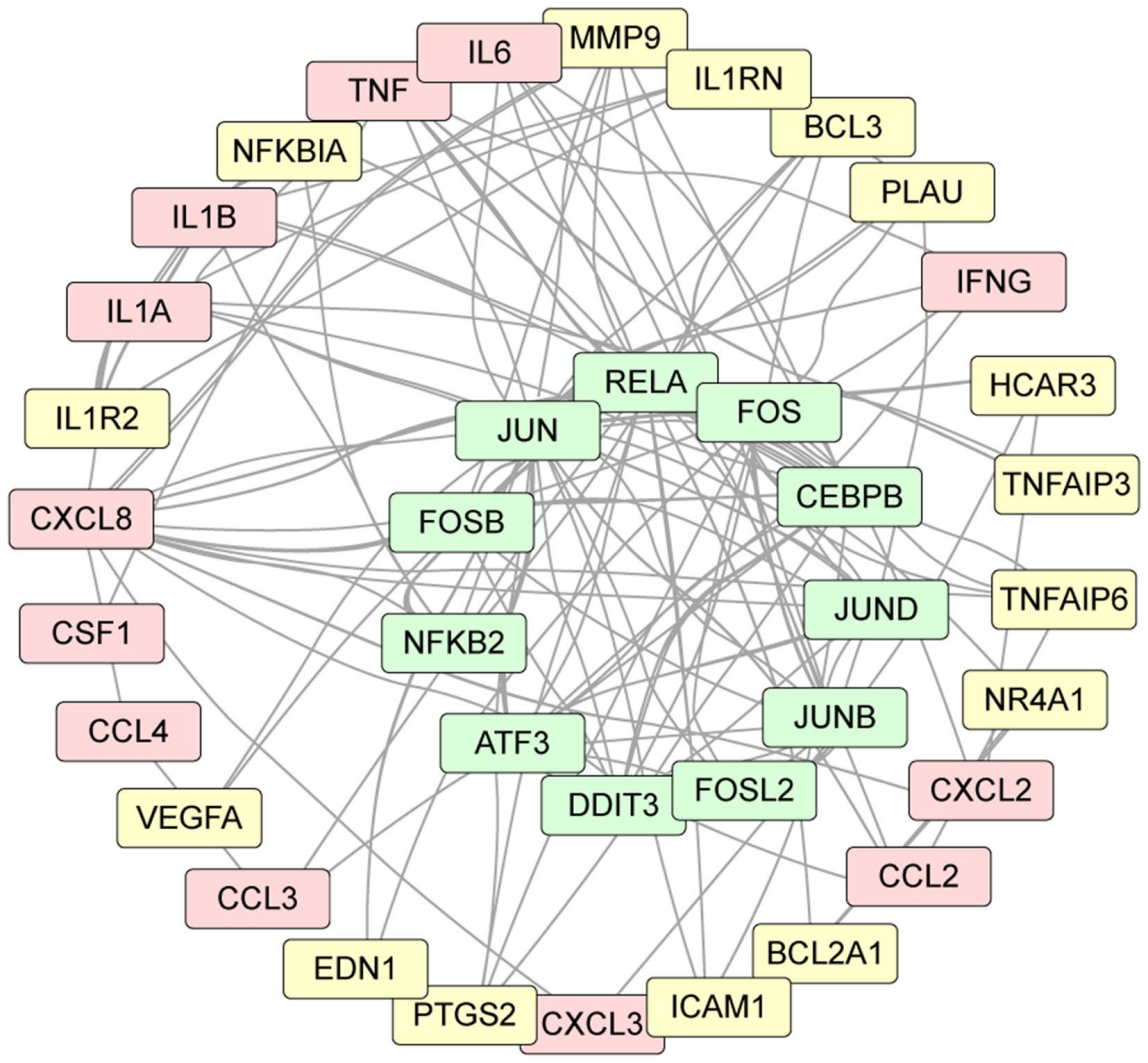
Overnight shipping induces an inflammatory transcriptional network in PBMCs. Weighted network analysis of transcripts differentially regulated in PBMCs isolated from blood held for 24 h relative to freshly prepared PBMCs revealed a highly interconnected network of 11 transcription factors (green) regulating 12 inflammatory cytokines and chemokines (red) and 15 additional inflammatory factors (yellow).

We assessed the transcriptional impact of holding blood at ambient temperature by isolating PBMCs and performing bulk RNA-seq (Supplemental Table 1). We compared the transcriptional profiles from 3 healthy donors (female age 29, female age 33, male age 52) in PBMCs prepared immediately after blood collection (0 h) and PBMCs prepared after holding the blood at ambient temperature for 24 h. Transcript read counts for 16,491 genes per subject per timepoint that passed initial quality control were analyzed by K-means clustering of the 2000 most variable genes (Fig. 3). Of the 4 clusters, only one (cluster D) exhibited uniform transcriptional changes in all 3 subjects, consistent with a gene set that was influenced specifically by the handling conditions rather than by inter-individual biological variations. The 695 genes in this cluster mapped to gene ontology biological processes and cellular components associated with blood cell development and function, adhesion and cellular movement, and inflammation. Relevant KEGG^15^ pathways involved cytokine and chemokine signaling. Analysis of differentially expressed genes (DEGs) in common across the three subjects using DEseq2^16^ on all 16,491 genes revealed that only 929 genes were upregulated more than twofold (FDR < 0.05) and only 215 genes were downregulated to the same extent (Supplemental Table 2; Supplemental Fig. 1). Weighted correlation network analysis^17^ further revealed 178 strongly regulated genes as part of a co-expression network (Table 3). Gene set enrichment analysis^18^ identified 10 enriched pathways, several of which were associated with inflammatory signaling (Table 4). Finally, weighted network analysis^19^ identified a highly interconnected network arising from 11 transcription factors regulating 12 inflammatory cytokines and chemokines (Fig. 4), including CXCL8, IL1α, IL1β, TNFα, and IL6.

**Table 3:** Transcriptional analysis: top coexpression module genes after 24 hours.

Gene	FI^1^	Gene	FI	Gene	FI	Gene	FI	Gene	FI	Gene	FI
CXCL8	140	CFAP45	11.9	LAMB3	7.9	NFE4	6.4	PANX2	5.6	NINJ1	4.6
ALPL	100	PLIN5	11.7	AOC3	7.9	ULK4P3	6.3	NEAT1	5.5	PHLDA1	4.6
CA4	74.2	MGAM	11.5	COL1A1	7.8	MIR222	6.2	LINC	5.5	FCAR	4.6
PLIN4	65.7	ATF3	11.4	IL1A	7.7	CCL3	6.2	LIMK2	5.5	FAM27B	4.5
PI3	45.1	C15orf48	11.4	PELI1	7.7	REM2	6.1	NR4A3	5.5	ATG2A	4.5
CNTNAP3	42.7	NECAB2	11.2	MAPK8IP1	7.6	DAPK2	6.1	MIR221	5.4	LOXL1	4.5
MME	42.5	FAM27C	11.1	JUND	7.5	KLF5	6.1	ITPRIP	5.4	HIF1A	4.5
G0S2	38.0	NIPAL4	11.0	MAFF	7.4	DUSP8	6.0	NBPF17P	5.3	MIR22HG	4.5
BTNL8	32.4	FOSB	10.4	FBXL13	7.4	MIR6835	6.0	F12	5.3	KLHL15	4.5
PLAU	28.1	ICAM1	10.4	TNFRSF10C	7.4	RPS26P43	6.0	PIM3	5.3	MIR6750	4.4
PHOSPHO1	21.1	LRG1	10.1	TRIB1	7.4	PPP1R15B	6.0	PLA1A	5.3	HSPA1B	4.4
HCAR2	20.2	HCAR3	10.0	ID1	7.2	PLAUR	6.0	HSPA2	5.3	MIR6821	4.4
CYP4F3	18.9	PLK3	9.6	CYP11A1	7.2	C17orf107	5.9	RGS1	5.3	PER1	4.4
CACNA1E	18.4	LINC00677	9.5	FAM71A	7.2	EDN1	5.9	IER5	5.2	SESN2	4.4
FCGR3B	16.7	MIR222HG	9.4	ZFP36	7.1	NAV3	5.9	AOC2	5.2	SUMO1P1	4.4
CCDC173	16.6	PROK2	9.4	BCL2A1	7.1	DUSP10	5.9	KLF6	5.2	RND1	4.4
CSF1	15.6	GADD45B	9.2	PHLDA2	7.1	TBCAP3	5.9	FFAR3	5.1	NXT1	4.4
MMP25	15.3	TNFAIP3	9.0	MIR612	6.9	ELL	5.9	MIDN	5.0	RGCC	4.4
PPP1R15A	15.2	CCN3	9.0	SFN	6.9	PIGA	5.9	KIAA0895	5.0	KCNK7	4.3
IL1R2	14.9	FFAR2	9.0	SNORD14E	6.8	LINC01002	5.9	LRRC70	5.0	SPATC1	4.3
DDIT3	14.4	CNTNAP3B	9.0	CXCR2	6.8	BCL3	5.8	PHACTR1	5.0	SPTLC1P1	4.2
MMP9	14.4	IFNG	8.9	KIAA0319	6.8	INSC	5.8	MANSC1	4.9	WNK4	4.2
CMTM2	14.4	CNTNAP3P2	8.7	UPB1	6.8	GCM1	5.8	HMGN1P3	4.9	IL6	4.1
NFKBIA	14.2	NSUN7	8.6	TRPM6	6.8	CEBPB	5.8	YPEL5	4.9	USP2	4.1
NAMPT	14.2	GADD45G	8.5	IL1RN	6.7	DNAAF1	5.8	CCNJL	4.9	C7orf61	4.1
NAMPTP1	14.0	DCST1	8.5	SLC25A37	6.6	ZDHHC18	5.7	MIR22	4.9	BTBD19	4.0
CSRNP1	13.4	PMAIP1	8.4	TNF	6.6	NFKBIZ	5.7	ROPN1L	4.8	MIR6775	4.0
KRT23	13.1	CHI3L1	8.3	VEGFA	6.6	CEACAM3	5.7	SOBP	4.8	IL1B	3.8
MIR616	12.3	ZC3H12A	8.3	GLIS2	6.5	MYL4	5.6	ZBTB10	4.7		
ADM	12.2	TNFAIP6	8.3	CD83	6.5	KREMEN1	5.6	CAMK1G	4.7		

^1^FI = fold induction.

**Table 4:** Gene set enrichment analysis: top upregulated pathways.

geneSet	Pathway	# Genes	FDR
hsa04657	IL-17 signaling pathway	12	7.67E-08
hsa04668	TNF signaling pathway	12	2.76E-07
hsa05323	Rheumatoid arthritis	11	2.76E-07
has04060	Cytokine-cytokine receptor interactions	13	2.1E-06
hsa04010	MAPK signaling pathway	16	6.27E-06
hsa05164	Influenza A	12	1.65E-05
hsa05202	Transcriptional misregulation in cancer	12	3.40E-05
hsa04064	NF-kappa B signaling pathway	9	3.40E-05
hsa04380	Osteoclast differentiation	10	3.76E-05
hsa04933	AGE-RAGE signaling pathway in diabetes	9	3.76E-05

### Peripheral blood mononuclear cell inflammatory responses are maintained after overnight shipping

**Figure 5:**
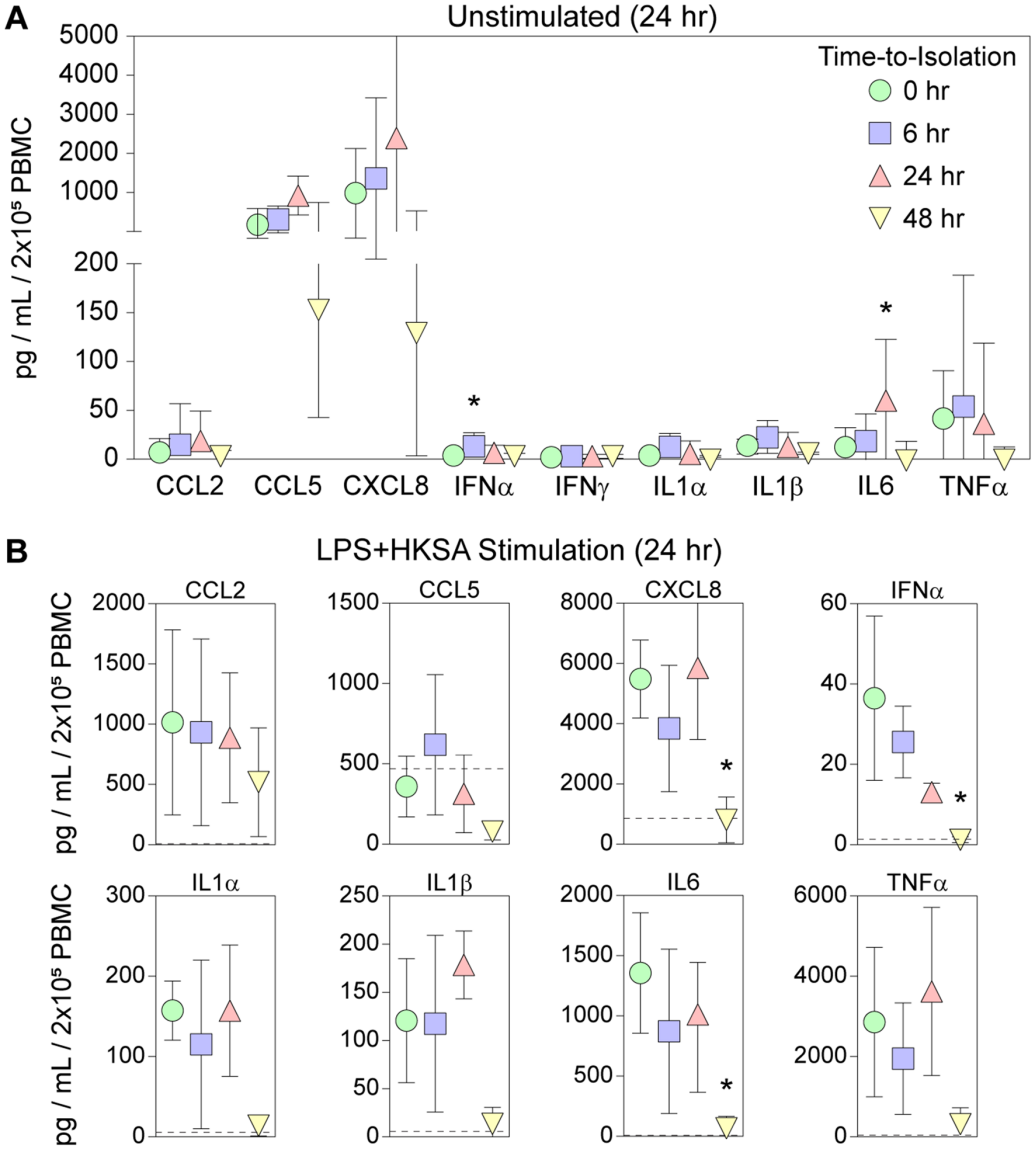
Spontaneous and stimulated release of inflammatory factors is maintained in PBMCs isolated from whole blood held at ambient temperature for 6 or 24 h. PBMCs isolated from blood held for 0, 6, 24, or 48 h were either incubated for 24 h in the absence of stimulation (**A**) or were incubated for 24 h with a cocktail of LPS and HKSA (**B**). Inflammatory cytokines and chemokines released into the supernatant were quantified by multiplex cytometric bead array. (**A**) Relative to PBMCs isolated from fresh blood, cells derived from blood held for 6 h at ambient temperature exhibited a small increase in spontaneous release of IFNα while cells held for 24 h released more IL6. (**B**) PBMCs from fresh blood stimulated for 24 h with LPS and HKSA released increased amounts of CCL2, CXCL8, IFNα, IL1α, IL1β, IL6, and TNFα. CCL5 release was not increased above spontaneous levels. This pattern was maintained in stimulated PBMCs isolated from blood held for 6 or 24 h at ambient temperature. However, PBMCs isolated from blood held for 48 h exhibited a uniform decrease in the stimulated response, with significant reductions in CXCL8, IFNα, and IL6 release. Symbols are color-coded as in the legend of (**A**). Analyte levels were normalized to pg per mL per 2 × 10^5^ PBMCs. Dashed lines in B represent the mean spontaneous (unstimulated) release level from the 0 h samples. Each symbol represents the mean of 3 individual donors and error bars show the 95% confidence intervals. Each analyte timecourse was assessed by one-way ANOVA; * indicates *P* < 0.05 vs 0 h by Dunnett’s pairwise comparison.

Ex vivo stimulation of immune cells is an important tool for assessing immune dysfunction, including both hypo- and hyperactivation phenotypes. Our standard approach to interrogating these responses is to gradient purify mononuclear cells from whole blood and incubate the cells for 24 h in RPMI containing 1% human serum and various pattern recognition receptor agonists. For this study we exposed cells to a pan-bacterial stimulus comprised of 1 µg/mL lipopolysaccharide (LPS) and 10^8^ copies/mL heat-killed *Staphylococcus aureus* (HKSA)^20^. We found that after 24 h at 37 °C, unstimulated gradient-purified mononuclear cells spontaneously released several cytokines and chemokines (Fig. 5A). Overall, these levels were neither increased nor decreased in cells isolated from whole blood held at ambient temperature for up to 48 h, though small but significant increases were observed in IFNα release from cells held for 6 h and in IL6 release from cells held for 24 h prior to PBMC isolation (Fig. 5A). Cells isolated from whole blood held at ambient for 48 h did show an apparent reduction in spontaneous release of CXCL8 and CCL5, but these changes did not reach significance. Of note, unstimulated cells isolated from blood held at ambient for 24 h did not spontaneously release more IFNα, IFNγ, IL1α, IL1β, or TNFα after 24 h at 37 °C, indicating that the ambient storage conditions did not induce an increase in release of these factors despite the transcriptional upregulation observed above. Finally, stimulation of purified mononuclear cells with LPS + HKSA for 24 h at 37 °C induced increased release of CCL2, CXCL8, IFNα, IL1α, IL1β, IL6, and TNFα. The amount of these factors released by the mononuclear cells was unaffected by ambient storage of the whole blood for 6 or 24 h prior to purification and stimulation (Fig. 5B). The stimulated response in mononuclear cells isolated from blood held for 48 h at ambient was almost uniformly decreased relative to fresh blood, consistent with the morphological degradation apparent in the cells at this timepoint (Figs. 1, 2), although only CXCL8, IFNα, and IL6 changes were significant.

### Neutrophil inflammatory responses are strongly degraded by overnight shipping

**Figure 6:**
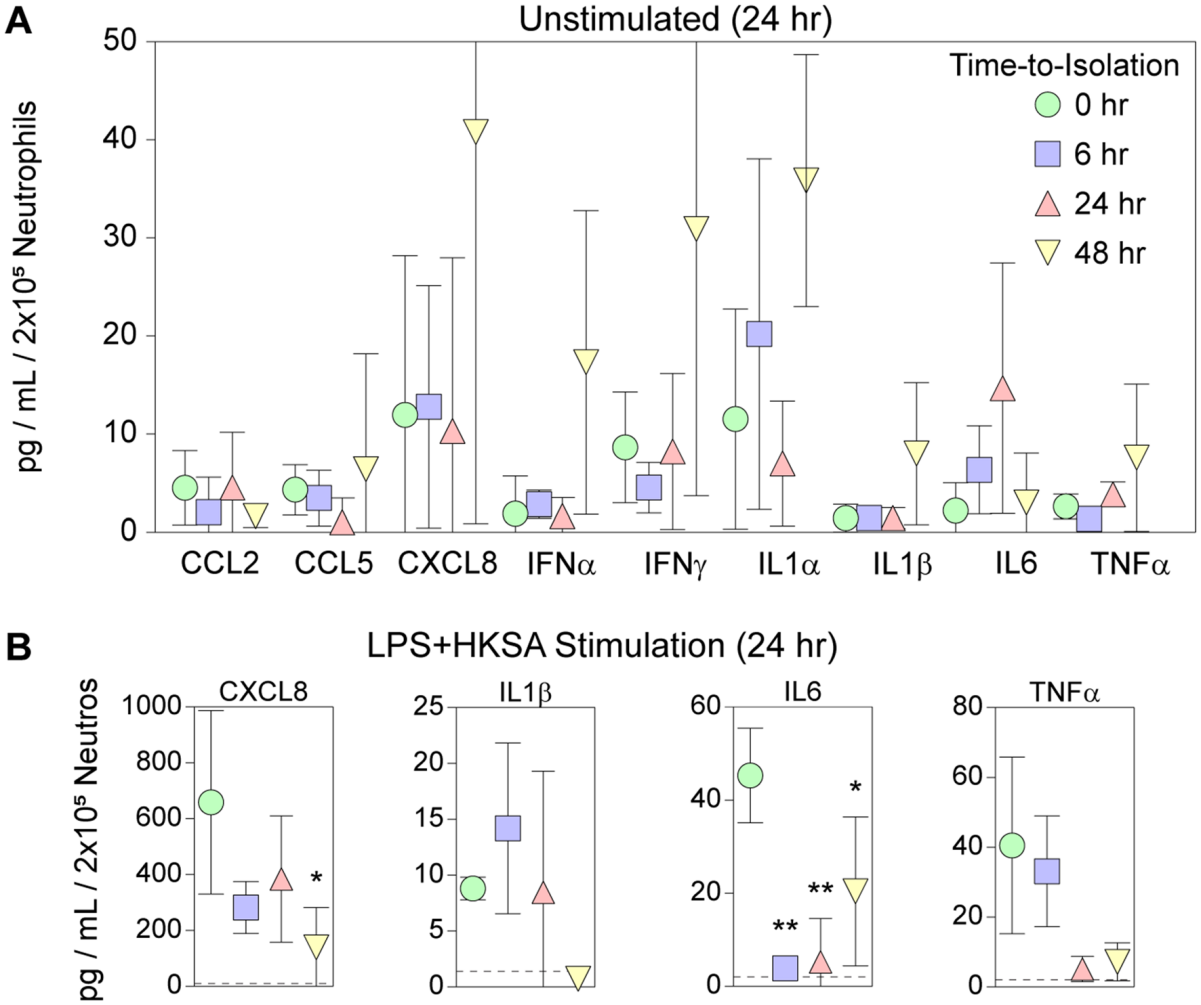
Spontaneous release of inflammatory factors is maintained in neutrophils from shipped whole blood but stimulated responses are quickly abrogated. Neutrophils isolated from blood held for 0, 6, 24, or 48 h were either incubated for 24 h in the absence of stimulation (**A**) or were incubated for 24 h with a cocktail of LPS and HKSA (**B**). Inflammatory cytokines and chemokines released into the supernatant were quantified by multiplex cytometric bead array. (**A**) Relative to neutrophils isolated from fresh blood, there were no outstanding changes in spontaneous release of factors from neutrophils isolated from shipped blood. (**B**) However, even neutrophils isolated from blood held for 6 h at ambient temperature showed loss of stimulated CXCL8, IL6, and TNFα release, though only CXCL8 in the 48 h group and IL6 in the 6, 24, and 48 h groups reached significance. CCL2, CCL5, IFNα, IFNγ, and IL1α were not released in response to LPS + HKSA in neutrophils from any timepoint. Symbols are color-coded as in the legend of (**A**). Each symbol represents the mean of 3 individual healthy donors; error bars show the 95% confidence limits. Analyte levels were normalized to pg per mL per 2 × 10^5^ neutrophils. Dashed lines in (**B**) represent spontaneous release level from the 0 h sample. Each analyte timecourse was assessed by one-way ANOVA; * indicates *P* < 0.05 vs 0 h and ** indicates *P* < 0.01 by Dunnett’s pairwise comparison.

Using the same approach as above, neutrophils isolated from fresh whole blood or blood that was held at ambient temperature for 6, 24, or 48 h were stimulated ex vivo with LPS + HKSA. While the unstimulated release of cytokines and chemokines was largely unaffected by ambient storage (Fig. 6A), the stimulated IL6 response was strongly attenuated even after only 6 h (Fig. 6B). The CXCL8 and IL1β responses were maintained in neutrophils derived from blood held overnight at ambient temperature but dropped to unstimulated levels in the 48 h group. The TNFα response was maintained in the 6 h group but dropped to unstimulated levels in the 24 h and 48 h groups, though these changes did not reach statistical significance due to the large variance among the 3 donors in neutrophils isolated from fresh blood. Overall, these findings are largely consistent with the altered morphology of the cells by the 48 h timepoint (Figs. 1, 2).

## Discussion

We modeled ambient temperature standard overnight delivery of whole blood collected in EDTA tubes in order to ascertain the impact of shipping on maintenance of PBMC responses. In addition to a 24 h timepoint that is compatible with point-to-point overnight delivery across the contiguous United States by such couriers as Federal Express, we included an early 6 h timepoint that models typical processing time at a local lab and a late 48 h timepoint that might occur with shipping delays. All analyses were compared to a “fresh” sample that was processed within approximately 20 min of phlebotomy. We found that the CBC/differential profile of the whole blood was largely unaffected by shipping time, even out to 48 h (Table 1). More sensitive analysis using flow cytometry revealed that overnight shipping led to physical changes in the white blood cells that manifested as forward and side scatter repositioning (Figs. 1, 2) and the migration of neutrophils into the lower density PBMC fraction (Fig. 2). Despite such physical changes, the immunophenotypic identification of lymphocytes, monocytes (including classical, nonclassical, and inflammatory subsets), and neutrophils remained largely intact following overnight shipping (Fig. 1). Analysis of the plasma fraction generated during isolation of PBMCs indicated stability in most inflammatory analytes (Table 2), suggesting that leukocytes sitting in whole blood at ambient temperatures over 24 h did not release large amounts of cytokines or chemokines. One caveat to this interpretation is that the endpoint levels of these factors may reflect a balance between ongoing degradation of factors present at the time of collection and spontaneous release of additional factors during shipping. The most notable exception was IL1β, the levels of which increased from undetectable in 3 of 5 subjects in fresh blood to readily detectable in the plasma of all subjects at 48 h. Given the inherent lability of IL1β, this finding suggests either that degradation of cytokines and chemokines in whole blood held at ambient temperature is insubstantial or that IL1β is uniquely over-produced relative to other inflammatory cytokines.

RNAseq analysis (Fig. 3) identified upregulation of a network involving 11 transcription factors associated with inflammation (Fig. 4) in PBMCs derived from blood shipped overnight. Weighted correlation analysis of the ~ 1000 genes upregulated at 24 h further refined the effect to a coexpression module comprised of 178 genes (Table 3). In parallel, gene set enrichment analysis of the coexpression module identified 10 key pathways associated with overnight shipping (Table 4). Of note, 7 of these pathways are dominated by the > 100-fold change in CXCL8 transcripts. The identification of TNFα signaling as a key pathway is contrasted by the absence of a detectable increase in TNFα in plasma from whole blood held at ambient temperature for 24 h (Table 2) (though see above regarding synthesis vs degradation). Ultimately, while it is certainly the case that 24 h shipping is associated with transcriptional changes in PBMCs, it is equally valid to note that of 16,491 genes analyzed, only ~ 1100 genes were either up- or down-regulated more than twofold, suggesting a relatively modest effect. Further support for this interpretation comes from the minor impact of overnight shipping on the levels of cytokines and chemokines spontaneously released by purified PBMCs incubated for an additional 24 h in RPMI containing 1% human serum (Fig. 5). Unstimulated PBMCs derived from blood held at ambient temperature for 24 h did not exhibit a substantial increase in basal release of most cytokines or chemokines relative to unstimulated PBMCs derived from fresh blood, with the exception of IL6, which increased from 12 pg/mL/2 × 10^5^ PBMCs to 55 pg/mL/2 × 10^5^ PBMCs. However, this change is very small compared to the scale of the induced IL6 response (Fig. 5B). Finally, these same PBMCs robustly responded to stimulation with LPS + HKSA by releasing cytokines and chemokines at levels comparable to the stimulated response in PBMCs derived from fresh whole blood (Fig. 5B). PBMCs isolated from blood held at ambient temperature for 24 h were statistically indistinguishable from PBMCs isolated from fresh blood. Overall, these findings support the maintenance of a stable phenotype in PBMCs isolated from whole blood shipped overnight at ambient temperatures and suggest that analysis of such responses in shipped blood provides an accurate representation of the cellular phenotype present in the patient at the moment of blood collection.

The application of precision medicine approaches to treat complex diseases mediated by polygenic, epigenetic, and environmental factors requires dynamic, reproducible, and quantitative biomarkers. The use of bulk population biomarkers such as the static level of serum proteins provides insufficient information for patient stratification or for tracking disease evolution and therapy response. Moreover, for rare diseases, each patient serves essentially as an “N of 1” and analysis of pathogenic mechanisms and therapeutic efficacy in such patients cannot depend upon bulk responses that are averaged across the total volume of the biospecimen or compared to the averaged responses of a control population. The study of perturbomics offers a solution to these problems^21^. The cellular perturbome consists of transcriptional, proteomic, secretomic, and metabolomic responses to ex vivo stimulation of specific cell types with pathogenically and/or therapeutically relevant agents. For many diseases and therapies, the most relevant perturbome is the response induced in peripheral blood mononuclear cells by stimulation with inflammatory drivers. For example, acquisition of a so-called “endotoxin tolerant” phenotype in monocytes derived from patients with sepsis or acute respiratory distress syndrome is marked by reduced TNFα production in response to ex vivo stimulation with LPS^22–24^. This monocyte anergy phenotype can be used to stratify patients and predict mortality^25^ and therapeutic restoration of function^26^.

A parallel issue that arises with the analysis of functional biomarkers, especially in the context of rare disease research, is the need to involve patients from multiple centers distributed over a large geographic area. For many rare diseases it would be impossible to study sufficient biospecimens at one site. Furthermore, while large tertiary care research institutions have the infrastructure and expertise necessary to process and freeze specimens, many relevant patients are seen at primary or secondary facilities that cannot coordinate complex specimen processing steps such as centrifugation. Thus, in order to capture these patients it is necessary to develop a process that facilitates shipping of an easy-to-collect specimen, such as whole blood in EDTA tubes. To further simplify the process, the receiving site can send out pre-made kits containing the necessary tubes and return shipping containers. For example, we routinely provide EDTA tubes, a Styrofoam tube holder within a hard plastic shipping container, gel packs (to reduce temperature fluctuations), and a Styrofoam box, along with prepaid FedEx labels. Our experience indicates that such kits facilitate participation by referring sites.

## Conclusions

Whole blood shipped overnight at ambient temperature provides a source of PBMCs that exhibit spontaneous and stimulated cytokine and chemokine responses that closely mirror those obtained from freshly drawn blood. This finding greatly increases the ability to enroll patients from widely distributed centers into studies measuring the pathogenic significance of altered PBMC responses to ex vivo stimulation. In particular, if control samples are processed under identical conditions, it is possible to effectively remove geography as a limiting factor in the enrollment of patients with rare diseases into studies involving analysis of functional immune responses.

## Methods

### Human subjects

All experiments were approved by the Mayo Clinic Institutional Review Board (#08–007846). Written informed consent was received from donors prior to the study. All methods were performed in accordance with the relevant guidelines and regulations. Six female and three male healthy control subjects between the ages of 25 and 52 were used for the experiments. The 3 subjects analyzed by RNAseq (Figs. 3, 4, Tables 3, 4) were also characterized in the ex vivo stimulation experiments (Figs. 5, 6) and were included in the complete blood counts and differential analysis along with other controls.

### Whole blood conditions

Whole blood was collected into 10 mL EDTA tubes (BD, 366,643) from healthy control subjects. Two tubes were assigned to each timepoint: 0, 6, 24 and 48 h. The “fresh” or 0 h tubes were delivered directly to the lab and processed within 20 min of venipuncture with no other handling. Other timepoints were exposed to a shipping analog to mimic how samples are sent and received within the lab. In brief, tubes were held in a Styrofoam plug (Great Northern Corporation, G065S060162) within a hard plastic mailer (Inmark Life Sciences, BM13SC16) and then placed into a 10-pound Styrofoam shipping container (Great Northern Corporation, 213377) along with gel packs and paper padding. Packages were roughly handled immediately after packaging, transported in the back of an SUV for approximately 120 min of drive time, then physically carried back into the lab and held until each appropriate processing timepoint.

### Complete blood count with differential

Whole blood was measured on a DxH 500 Hematology Analyzer (Beckman Coulter, B40601) to obtain complete blood counts and leukocyte differential. Each day before use, the instrument was calibrated using control blood (Beckman Coulter, B36872) to validate detection ranges. Whole blood was extracted directly from the EDTA tube into the machine. Normal ranges are those established by the Mayo Clinic Laboratories^27^.

### Whole blood immunophenotyping

Starting with 300 µL of whole blood, 100 µL was transferred to a 1.5 mL microfuge tube and designated for the 1° cocktail. Antibodies in the final cocktail were FITC anti-CD16 (Biolegend, 302006), PE anti-CD14 (Tonbo, 50–0149-T100), PerCPCy5.5 anti-CD3 (Biolegend, 300,328), APC anti-HLADR (Biolegend, 307610) and AlexaFluor700 anti-CD66b (Biolegend, 305114). In addition to 6 single stain controls a fluorescence minus one (FMO) control was used that excluded APC anti-HLADR from the cocktail. Control samples (25 µL whole blood) were brought to 100 µL with room temperature DPBS (Corning, 21-031-CV). All tubes received 25 µL of normal mouse serum (Jackson ImmunoResearch, 015–000-120) to block non-specific sites. After blocking for 10–15 min, 5 µL of each primary antibody was added to the respective tube (~ 1:25 dilution). Samples were incubated at room temperature for 20 min protected from light and then 1 mL of VersaLyse (Beckman Coulter, A09777) was added to all tubes, mixed by gentle vortexing, and incubated at room temperature for 20 min protected from light. Tubes were centrifuged at 400 g for 3 min at room temperature and the supernatant was carefully aspirated down to the pellet. Cells were resuspended in 1 mL of room temperature PBS containing 1% BSA (SeraCare, 1900–0016). Tubes were centrifuged again at 400 g for 3 min at room temperature and the supernatant was carefully aspirated. Cells were resuspended in 300 µL of ice-cold freshly prepared fixative containing 0.2% paraformaldehyde (Sigma, 158127) in PBS. Cells were held on ice and protected from light until flow cytometric analysis.

### Peripheral blood mononuclear cell (PBMC) isolation

Leucosep tubes (Greiner Bio-One, 163290P) were used to prepare PBMCs. Each 12 mL tube can hold 3 to 8 mL of whole blood. Tubes were loaded with 3 mL of room temperature Lymphoprep (STEMCELL Technologies, 7801) and centrifuged at 800 g for 30 s to push Lymphoprep through filter. Approximately 7 mL of whole blood was decanted directly from the EDTA tube into each Lymphoprep tube and centrifuged at 800 g for 15 min at 20 °C in a swinging bucket rotor with no brake. Plasma was collected off the top fraction without disturbing the cell layer, aliquoted, and stored at − 80 °C. The PBMC-enriched fraction from the Leucosep tube was added to 40 mL of room temperature RPMI (Invitrogen, 11875–093) in a 50 mL conical using a 1 mL pipet. The final volume was brought to 50 mL with RPMI and the tube was gently inverted to mix, then centrifuged at 250 g for 10 min at 20 °C in a swinging bucket rotor. The supernatant was aspirated and the cell pellet was resuspended in 5 mL of RPMI containing 1% human serum (Sigma, H6914). Cells were counted and plated at 2 × 10^5^ cells per well in 200 µL in ultra-low attachment U-bottom 96-well plates (Corning, 7007) for ex vivo stimulation.

### Neutrophil isolation

Neutrophils were isolated from whole blood using the EasySep Direct Human Neutrophil Isolation Kit (STEMCELL Technologies, 19,666). 100 µL of isolation cocktail was added to each polystyrene round bottom tube (Falcon, 352054) containing 2 mL whole blood and samples were incubated at room temperature for 5 min. RapidSpheres were vortexed for 30 s and 80 µL was added to each sample. After 3 min at room temperature the volume was adjusted to 2.5 mL with room temperature DPBS (Corning, 21-031-CV) containing 1 mM EDTA (Invitrogen, 15575020). Samples were gently mixed by pipetting up and down 2–3 times and immediately placed (without lids) into an EasySep magnet (STEMCELL Technologies, 18000) for 3 min at room temperature. Using one continuous motion, the enriched cell suspension was decanted into a new polystyrene tube. The sample was immediately placed back into the EasySep magnet and the process was repeated. The final suspension was brought to 40 mL with room temperature DPBS and centrifuged at 400 g for 10 min. The supernatant was aspirated the cell pellet was resuspended in 5 mL of ex vivo stimulation media. Neutrophils were counted and plated at 2 × 10^5^ cells per well in 200 µL in ultra-low attachment U-bottom 96-well plates (Corning, 7007) for ex vivo stimulation.

### Ex vivo stimulation

Isolated cells from 3 individual donors were stimulated with a pan-bacterial cocktail comprised of lipopolysaccharide from *Escherichia coli* O26:B6 (LPS) (Sigma, L2654) at 1 µg/mL and heat-killed *Staphylococcus aureus* (HKSA) (InvivoGen, tlrl-hksa) at 10^8^ cells/mL. Commercial stocks were reconstituted at 100 × in UltraPure™ DNase/RNase-Free Distilled Water (Invitrogen, 10977015) and stored as individual use aliquots at − 20 °C. Unstimulated cells received 2 µL water as vehicle. Cells were incubated for 24 h in RPMI containing 1% human serum plus the relevant stimulus at 37 °C with gentle agitation at 80 rpm on an orbital shaker (Eppendorf, M1190-0000) housed inside a standard tissue culture incubator (5% CO_2_, humidified). At endpoint, cells were collected by centrifugation at 400 g for 5 min at room temperature and supernatants were collected, clarified at > 16000 g for 5 min, and stored at − 20 °C.

### Multiplexed cytometric bead array analysis

Supernatants (50 µL) from stimulated cells were analyzed using the Human Soluble Protein Master Buffer Kit (BD, 558264) and cytometric beads reactive for IL-6 (BD, 558276), CXCL8 (BD, 558277), IL1β (BD, 558279), IFNα (BD, 560379), CCL5 (BD, 558324), IL1α (BD, 560153), CCL2 (BD, 558287), TNFα (BD, 558273), and IFNγ (BD, 558,269). Plasma samples (50 µL) were analyzed using the Human Inflammatory Cytokine kit (BD, 551811) (CXCL8, IL1β, IL6, IL10, IL12p70, TNFα) and the Human Chemokine kit (BD, 552,990) (CCL2, CCL5, CXCL8, CXCL9, CXCL10).

### RNA sequencing

RNA was isolated from PBMCs using the RNeasy Mini Kit (Qiagen, 74106) following manufacturer instructions. Optional DNase digestion steps were performed and RNA was eluted into 50 µL of RNAse-free water. Initial RNA concentrations were obtained via Qubit RNA BR (Broad-Range) Assay Kit (Qubit, Q10210). Concentration and RNA integrity number were determined using the Agilent Fragment Analyzer system (Supplemental Table 1). RNAseq analysis was performed by the Mayo Clinic Genome Analysis Core. In brief, libraries were prepared from 200 ng input RNA using Illumina TruSeq RNA Access and then sequenced on an Illumina NovaSeq 6000. Paired-end reads at 2 × 150 bp were performed on all samples with a minimum read depth of 56 M reads and a minimum of 81% mapped reads (Supplemental Table 1). All sequences were assessed using MultiQC prior to downstream processing (Supplemental Table 1; Supplemental Fig. 1A). Results were processed via the MAPRSEQ pipeline^28^ to generate alignments and normalized gene counts that were further post-processed in iDEP.94^29^. Briefly, RSeQC^30^ was used for quality control of sequencing reads and the STAR aligner was used to generate alignment files. Normalized FPKM values for gene counts were obtained using featureCounts^31^ in the Rsubread package^32^. Genes with CPM value less than 1 across all samples were filtered out of subsequent analysis. Normalized FPKM values were log_2_ transformed with edgeR^33,34^ and k-Means clustering^35^ was performed on the 2000 most variable genes using the GeneOntology biological process and cellular component databases^36^ and the KEGG pathway database^37^ to identify non-redundant genesets. Differentially expressed genes were identified using DESeq2^16^ with a maximum FDR of 0.05 and a minimum twofold change in expression between groups (Supplemental Table 2; Supplemental Fig. 1B). Gene set enrichment analysis was performed in GSEA 4.2.3^18^ and weighted correlation network analysis was performed with the WGCNA package^17^ within iDEP using default settings (1000 most variable genes; soft threshold = 5; minimum module size = 20 genes; edge threshold = 0.4).

### Data analysis

Flow cytometric data were analyzed in FlowJo v10.8.1 (BD). Cytometric bead array data were preliminarily analyzed using FCAP Array (BD) and then reprocessed manually using FlowJo and standard curve fitting in Prism 9.4.0 (GraphPad). Statistics were generated in Prism 9.4.0. Quantitative metrics were graphed in Prism and then modified in Adobe Illustrator for layout and consistency.

## Supplementary Information

Supplementary Information 1.

Supplementary Information 2.

Supplementary Information 3.

Supplementary Information 4.

## Data Availability

The datasets used and/or analyzed during the current study are available from the corresponding author on reasonable request.
