# Supplementary figures and images for: Peripheral blood mononuclear cell phenotype and function are maintained after overnight shipping of whole blood
Source: Sci Rep. 2022 Nov 19;12:19920. doi: 10.1038/s41598-022-24550-6 (PMC9675784; doi:10.1038/s41598-022-24550-6)

**A**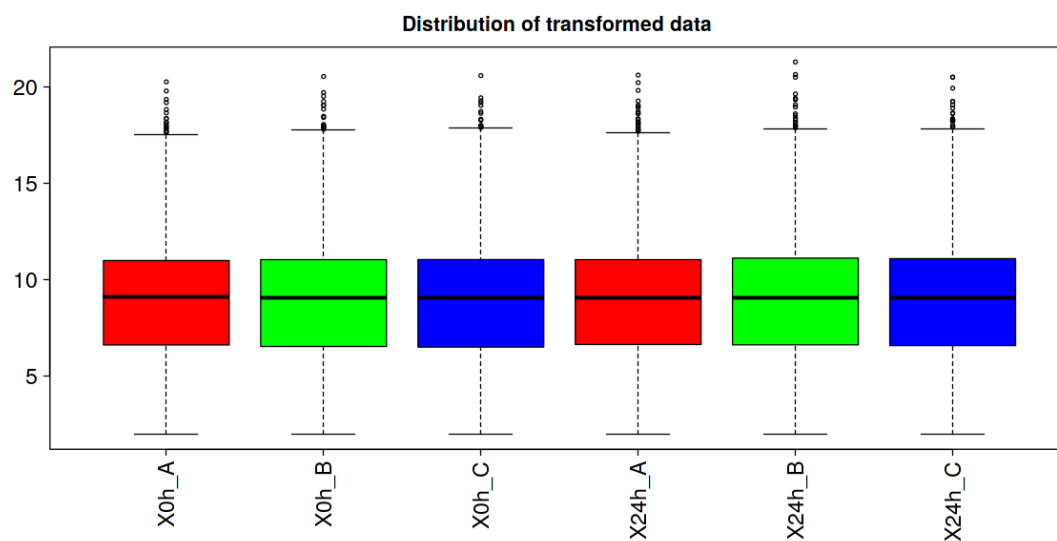**B**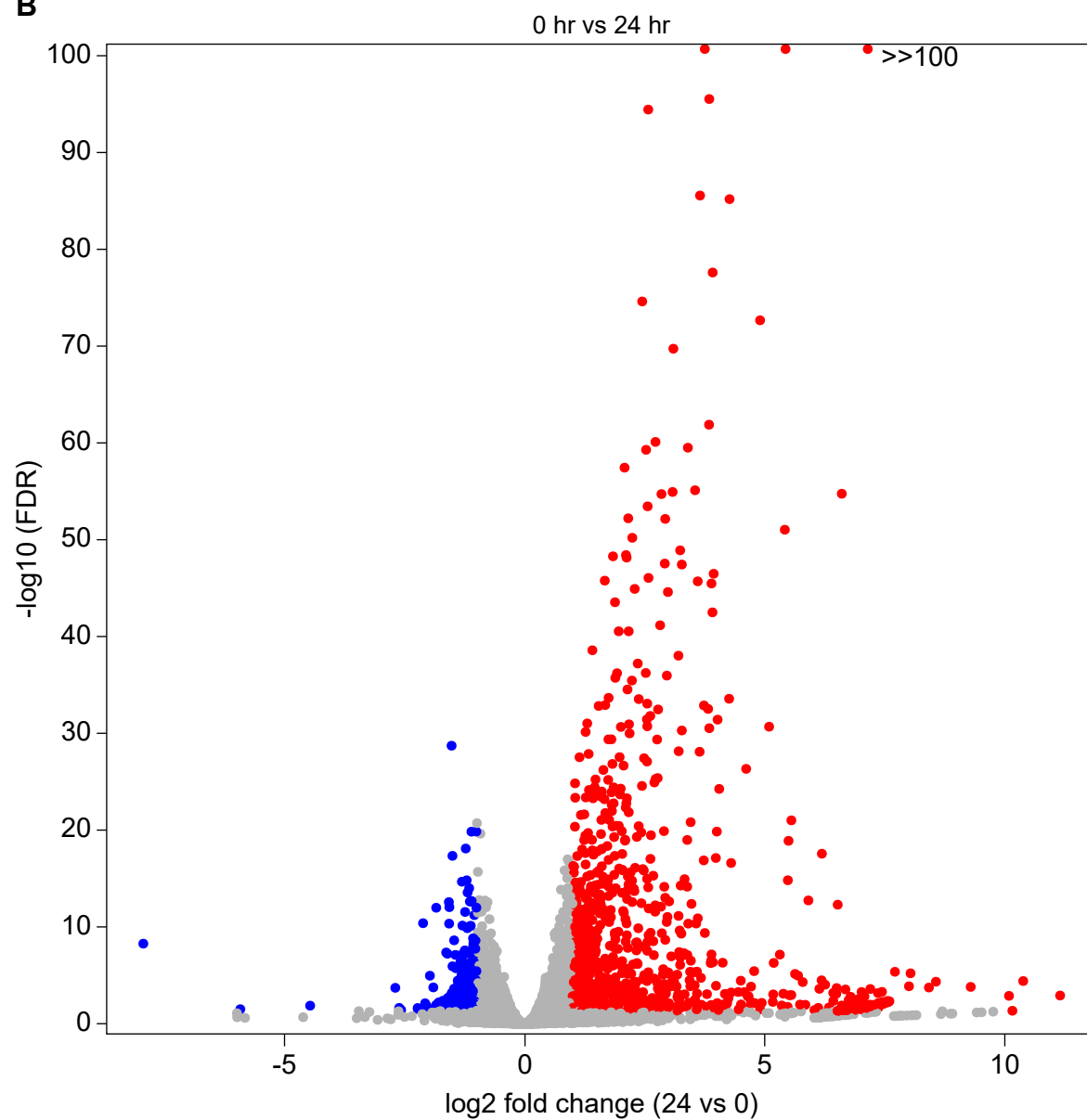

Supplement: Supplementary file 1 — Supplementary Information 1. [file 41598_2022_24550_MOESM1_ESM.pdf]
