# Supplementary material for: Peripheral blood mononuclear cell phenotype and function are maintained after overnight shipping of whole blood
Source: Sci Rep. 2022 Nov 19;12:19920. doi: 10.1038/s41598-022-24550-6 (PMC9675784; doi:10.1038/s41598-022-24550-6)
Supplement: Supplementary file 2 — Supplementary Information 2. [file 41598_2022_24550_MOESM2_ESM.pdf]

Supplemental Table 1. RNAseq Quality Control

Sample	Total RNA	RIN[1]	Total Reads	Mapped Reads	Junctions	Gene Count
Subject A 0 hr	3654 ng	9.9	76,741,630	85%	94%	75%
Subject B 0 hr	2234 ng	9.7	56,152,546	82%	87%	72%
Subject C 0 hr	5913 ng	9.8	86,673,600	84%	100%	77%
Subject A 24 hr	6937 ng	8.5	68,305,028	83%	92%	74%
Subject B 24 hr	5456 ng	8.6	63,322,326	81%	96%	73%
Subject C 24 hr	2490 ng	8.1	58,655,495	85%	100%	77%

[1]RNA Integrity Number.
