# Supplementary material for: Peripheral blood mononuclear cell phenotype and function are maintained after overnight shipping of whole blood
Source: Sci Rep. 2022 Nov 19;12:19920. doi: 10.1038/s41598-022-24550-6 (PMC9675784; doi:10.1038/s41598-022-24550-6)
Supplement: Supplementary file 3 — Supplementary Information 3. [file 41598_2022_24550_MOESM3_ESM.pdf]

Supplemental Table 2. Differentially Expressed Genes

Gene	Symbol	24 hr vs 0 hr log2 FC	FDR
ENSG00000274404	GOLGA6L22	11.15	0.001227
RP11-483E23.2	NA	10.38	3.92E-05
ENSG00000174450	GOLGA6L2	10.16	0.045819
RP11-566K19.9	NA	10.09	0.001331
ENSG00000163395	IGFN1	9.29	0.000158
ENSG00000117983	MUC5B	8.57	4.60E-05
ENSG00000170927	PKHD1	8.42	0.000184
C19ORF45	NA	8.04	6.21E-06
ENSG00000081479	LRP2	8.00	0.000136
ENSG00000154529	CNTNAP3B	7.71	4.33E-06
SPANXB2	NA	7.60	0.004777
ENSG00000204961	PCDHA9	7.58	0.005591
ENSG00000181984	GOLGA8CP	7.56	0.004391
ENSG00000150893	FREM2	7.55	0.006407
ENSG00000248640	HNRNPA1P56	7.52	0.00626
ENSG00000277518	MUC6	7.48	0.00864
RP11-611L7.3	NA	7.46	0.005591
ENSG00000009950	MLXIPL	7.44	0.000514
ENSG00000186832	KRT16	7.44	0.010042
ENSG00000060718	COL11A1	7.42	0.010429
ENSG00000133710	SPINK5	7.38	0.007255
ENSG00000276420	PRAMEF5	7.37	0.021418
ENSG00000282664	LINC00552	7.37	0.007522
ENSG00000152591	DSPP	7.36	0.000686
ENSG00000165837	ERICH6B	7.28	0.011199
ENSG00000200564	SNORD115-39	7.20	0.011138
ENSG00000124467	PSG8	7.20	0.016088
ENSG00000018236	CNTN1	7.20	0.00026
ENSG00000189064	GAGE2A	7.18	0.011199
ENSG00000186442	KRT3	7.15	0.012688
ENSG00000169429	CXCL8	7.15	7.42E-283
ENSG00000187984	ANKRD19P	7.12	0.011142
ENSG00000038295	TLL1	7.12	0.022922
ENSG00000180777	ANKRD30B	7.12	0.022922
ENSG00000288318	PALM3	7.11	0.018294
ENSG00000160868	CYP3A4	7.08	0.001525
ENSG00000177910	SPATA31C2	7.06	0.015753
ENSG00000148513	ANKRD30A	7.06	0.01759
ENSG00000103546	SLC6A2	7.03	0.015881
ENSG00000171124	FUT3	7.02	0.000528
ENSG00000236459	HNRNPA1P22	6.99	0.01669
ENSG00000100170	SLC5A1	6.98	0.032125
ENSG00000111245	MYL2	6.98	0.00671
ENSG00000230246	SPATA31C1	6.96	0.020493
RP11-400G3.5	NA	6.96	0.004801
ENSG00000042781	USH2A	6.95	0.025658
ENSG00000105929	ATP6V0A4	6.95	0.00334
ENSG00000144452	ABCA12	6.93	0.003162
AC005754.7	NA	6.89	0.0228
ENSG00000090402	SI	6.88	0.033634
ENSG00000112319	EYA4	6.88	0.02759
ENSG00000170289	CNGB3	6.87	0.038208
ENSG00000092054	MYH7	6.86	0.026022
ENSG00000204478	PRAMEF20	6.86	0.023117
RP11-1236K1.8	NA	6.83	0.024394
ENSG00000165841	CYP2C19	6.82	0.00738
ENSG00000008118	CAMK1G	6.79	0.001665
ENSG00000215771	LRRC37A14P	6.76	0.003696
ENSG00000201679	SNORD115-15	6.76	0.027957
ENSG00000177839	PCDHB9	6.75	0.003492
ENSG00000150244	TRIM48	6.74	0.006501
AC006995.8	NA	6.72	0.032397
ENSG00000243130	PSG11	6.71	0.040806
ENSG00000077498	TYR	6.70	0.035593

ENSG00000104321	TRPA1	6.69	0.039948
RP11-737O24.2	NA	6.68	0.000292
ENSG00000138347	MYPN	6.68	0.033608
ENSG00000171759	PAH	6.67	0.037574
ENSG00000170454	KRT75	6.63	0.041818
ENSG00000003137	CYP26B1	6.62	0.040722
ENSG00000253119	HNRNPA3P7	6.61	0.016605
RP11-367J7.3	NA	6.61	0.013162
ENSG00000167434	CA4	6.61	1.78E-55
ENSG00000197408	CYP2B6	6.52	0.049479
ENSG00000162551	ALPL	6.52	5.09E-13
ENSG00000199782	SNORD115-9	6.52	0.047019
ENSG00000162771	FAM71A	6.50	0.000207
RP11-157L3.10	NA	6.49	0.000804
ENSG00000205277	MUC12	6.43	0.004412
ENSG00000281151	KCNQ2	6.43	0.000657
ENSG00000154415	PPP1R3A	6.34	0.020802
ENSG00000168903	BTNL3	6.26	9.30E-05
ENSG00000124102	PI3	6.19	2.75E-18
ENSG00000118785	SPP1	6.18	3.29E-05
ENSG00000170092	SPDYE5	6.14	0.000251
ENSG00000109061	MYH1	6.12	0.023187
ENSG00000274391	TPTE	6.11	0.034894
ENSG00000255408	PCDHA3	6.04	0.03905
ENSG00000167676	PLIN4	5.91	1.84E-13
ENSG00000164796	CSMD3	5.85	0.011203
ENSG00000039139	DNAH5	5.79	5.02E-05
ENSG00000113248	PCDHB15	5.74	0.010538
ENSG00000125780	TGM3	5.69	1.09E-05
ENSG00000270069	MIR222HG	5.63	7.21E-06
ENSG00000196549	MME	5.56	9.80E-22
ENSG00000283876	MIR4260	5.55	0.001088
ENSG00000230257	NFE4	5.52	0.00115
ENSG00000113303	BTNL8	5.50	1.28E-19
ENSG00000173868	PHOSPHO1	5.48	1.52E-15
ENSG00000139219	COL2A1	5.48	0.018132
ENSG00000123689	G0S2	5.43	5.62E-123
ENSG00000106714	CNTNAP3	5.42	9.30E-52
RP11-157L3.5	NA	5.39	0.030385
RP11-290L7.3	NA	5.39	0.000799
ENSG00000181358	CTAGE10P	5.35	0.013043
ENSG00000231527	FAM27C	5.32	7.29E-08
ENSG00000214456	PLIN5	5.19	5.25E-07
ENSG00000140478	GOLGA6D	5.18	0.000936
ENSG00000136206	SPDYE1	5.17	0.005591
ENSG00000122861	PLAU	5.09	2.07E-31
RP11-526D8.7	NA	4.94	0.007027
GPR97	NA	4.90	2.15E-73
ENSG00000261812	TUBB8P7	4.90	0.033906
ENSG00000180210	F2	4.80	0.007489
AC116366.6	NA	4.78	3.71E-06
ENSG00000167195	GOLGA6C	4.72	0.005591
ENSG00000157335	CLEC18C	4.72	0.000148
ENSG00000125414	MYH2	4.67	0.049723
ENSG00000244641	RPS26P43	4.66	0.001458
ENSG00000276386	CNTNAP3P2	4.65	0.001481
ENSG00000198216	CACNA1E	4.61	4.69E-27
ENSG00000229145	ACTBP1	4.58	0.026564
AC051649.12	NA	4.58	0.000537
ENSG00000101680	LAMA1	4.52	0.014315
ENSG00000283022	HYDIN	4.51	0.010205
RP11-68I3.11	NA	4.50	3.60E-05
ENSG00000244411	KRTAP5-7	4.49	0.033616
ENSG00000278598	MIR6775	4.46	0.03288
ENSG00000282509	ULK4P3	4.45	0.000532
ENSG00000200879	SNORD14E	4.30	0.000585

ENSG00000154479	CCDC173	4.30	2.51E-17
ENSG00000186529	CYP4F3	4.27	6.55E-86
ENSG00000182782	HCAR2	4.26	2.70E-34
ENSG00000207725	MIR222	4.24	0.001702
ENSG00000188487	INSC	4.24	0.002593
ENSG00000274986	MIR6750	4.22	0.008161
ENSG00000207870	MIR221	4.17	0.000827
ENSG00000164683	HEY1	4.14	0.012038
ENSG00000237975	FLG-AS1	4.14	0.010234
ENSG00000130222	GADD45G	4.12	5.03E-07
ENSG00000162747	FCGR3B	4.05	5.56E-25
RP11-363E6.3	NA	4.04	0.037898
ENSG00000184371	CSF1	4.02	3.87E-32
RP11-727A23.8	NA	4.01	0.007345
RP11-1220K2.2	NA	4.00	1.41E-20
ENSG00000259717	LINC00677	3.99	0.000121
ENSG00000008516	MMP25	3.98	7.60E-18
ENSG00000166592	RRAD	3.95	0.023417
ENSG00000115590	IL1R2	3.94	3.29E-47
RP11-95M15.2	NA	3.92	4.48E-07
ENSG00000087074	PPP1R15A	3.92	2.49E-78
ENSG00000175197	DDIT3	3.91	3.19E-43
ENSG00000163357	DCST1	3.89	7.01E-08
ENSG00000229644	NAMPTP1	3.89	3.35E-46
ENSG00000267858	MZF1-AS1	3.89	0.011535
NOV	NA	3.89	3.08E-07
RP11-694O4.1	NA	3.88	5.43E-07
ENSG00000100985	MMP9	3.85	2.96E-31
ENSG00000100906	NFKBIA	3.85	2.98E-96
ENSG00000105835	NAMPT	3.84	1.31E-62
ENSG00000263309	KRT23	3.82	3.02E-33
RP13-452N2.1	NA	3.80	0.00089
RP11-166O4.1	NA	3.77	0.017805
CTD-3088G3.8	NA	3.75	4.27E-10
ENSG00000144655	CSRNP1	3.75	2.28E-213
ENSG00000140279	DUOX2	3.74	0.01867
ENSG00000148926	ADM	3.73	1.33E-33
ENSG00000140932	CMTM2	3.73	1.34E-17
RP11-974F13.5	NA	3.68	0.01097
ENSG00000103154	NECAB2	3.67	1.85E-05
ENSG00000208028	MIR616	3.65	2.77E-86
ENSG00000213085	CFAP45	3.64	8.05E-29
ENSG00000166920	C15orf48	3.61	1.30E-11
ENSG00000177606	JUN	3.61	1.99E-46
RP11-372K14.2	NA	3.60	0.017292
ENSG00000179571	NBPF17P	3.59	0.000766
RP11-39C10.1	NA	3.59	4.18E-06
ENSG00000205592	MUC19	3.58	0.032532
ENSG00000163993	S100P	3.58	0.008906
CTB-50L17.14	NA	3.57	4.49E-11
ENSG00000162772	ATF3	3.55	7.77E-56
ENSG00000175793	SFN	3.55	0.003257
ENSG00000172548	NIPAL4	3.54	0.000106
ENSG00000115008	IL1A	3.53	4.90E-06
ENSG00000236438	FAM157A	3.48	4.19E-13
ENSG00000185022	MAFF	3.46	3.14E-07
ENSG00000125740	FOSB	3.46	1.51E-21
ENSG00000288362	CYP11A1	3.45	0.000136
ENSG00000131471	AOC3	3.45	4.29E-06
ENSG00000274538	PHLDA2	3.44	3.26E-05
ENSG00000274636	GLIS2	3.42	0.00666
FLJ45079	NA	3.41	4.58E-11
ENSG00000090339	ICAM1	3.40	3.12E-60
ENSG00000144837	PLA1A	3.39	0.012257
RP11-334L9.1	NA	3.39	8.12E-08
ENSG00000282607	MGAM	3.39	1.04E-19

ENSG00000179299	NSUN7	3.39	7.01E-15
ENSG00000248323	LUCAT1	3.35	0.016337
ENSG00000171236	LRG1	3.33	1.17E-15
ENSG00000137270	GCM1	3.33	0.000923
ENSG00000044574	HSPA5	3.32	5.17E-08
ENSG00000255398	HCAR3	3.27	5.10E-31
ENSG00000173846	PLK3	3.27	3.66E-48
ENSG00000067798	NAV3	3.27	0.020564
ENSG00000108821	COL1A1	3.26	4.98E-10
ENSG00000111537	IFNG	3.25	5.35E-15
KIAA1257	NA	3.24	1.41E-07
ENSG00000099860	GADD45B	3.24	1.25E-49
ENSG00000137261	KIAA0319	3.24	8.40E-07
AC084368.1	NA	3.23	0.015117
ENSG00000121653	MAPK8IP1	3.23	2.36E-10
ENSG00000241721	SUMO1P1	3.22	0.003259
ENSG00000276753	MIR6821	3.22	0.002979
ENSG00000125968	ID1	3.22	3.98E-08
ENSG00000273822	MUC4	3.21	0.00938
ENSG00000126262	FFAR2	3.21	7.11E-29
AC093495.4	NA	3.21	7.35E-12
ENSG00000118503	TNFAIP3	3.20	9.57E-39
ENSG00000073150	PANX2	3.20	0.000875
ENSG00000285479	CACNA1C	3.18	0.045534
RP11-779O18.2	NA	3.18	0.001845
RP11-190A12.7	NA	3.18	8.56E-06
ENSG00000102010	BMX	3.16	0.00014
ENSG00000189337	KAZN	3.13	0.024277
ENSG00000249055	TBCAP3	3.11	4.30E-05
ENSG00000141682	PMAIP1	3.10	1.86E-70
ENSG00000188290	HES4	3.10	0.000129
ENSG00000163874	ZC3H12A	3.08	1.17E-55
ENSG00000123610	TNFAIP6	3.05	1.10E-07
ENSG00000214249	CTAGE11P	3.05	5.93E-05
ENSG00000231721	LINC-PINT	3.01	7.29E-06
ENSG00000229314	ORM1	3.01	2.34E-13
ENSG00000259699	HMGB1P8	3.01	0.007656
ENSG00000163421	PROK2	3.01	0.000159
RP11-1228E12.1	NA	2.99	0.016131
ENSG00000125845	BMP2	2.99	0.019797
ENSG00000175592	FOSL1	2.99	4.91E-06
ENSG00000124731	TREM1	2.98	2.55E-45
ENSG00000161040	FBXL13	2.97	9.38E-12
ENSG00000126562	WNK4	2.97	0.016791
ENSG00000130522	JUND	2.96	1.09E-36
ENSG00000133048	CHI3L1	2.96	3.09E-11
ENSG00000130383	FUT5	2.94	0.000784
AC012066.1	NA	2.93	0.016463
CTD-2262B20.1	NA	2.93	2.18E-09
ENSG00000283791	MIR612	2.93	9.73E-14
ENSG00000197329	PELI1	2.93	7.08E-53
ENSG00000173535	TNFRSF10C	2.92	2.92E-48
RP11-126K1.2	NA	2.92	0.005478
ENSG00000196878	LAMB3	2.91	7.16E-15
ENSG00000154099	DNAAF1	2.91	0.001085
ENSG00000173334	TRIB1	2.90	1.28E-20
ENSG00000124107	SLPI	2.90	7.84E-05
RP11-206L10.1	NA	2.89	2.31E-06
ENSG00000158089	GALNT14	2.88	0.000698
RP13-516M14.8	NA	2.88	8.56E-06
RP3-342P20.2	NA	2.88	0.024844
ENSG00000145362	ANK2	2.86	0.013323
ENSG00000100024	UPB1	2.86	4.48E-10
ENSG00000128016	ZFP36	2.85	1.94E-55
ENSG00000172602	RND1	2.85	0.001473
RP11-519G16.3	NA	2.84	0.001373

ENSG00000157551	KCNJ15	2.84	8.13E-13
ENSG00000217783	LDHAL6FP	2.83	0.01887
ENSG00000140379	BCL2A1	2.82	6.87E-42
ENSG00000112320	SOBP	2.80	0.000288
ENSG00000135245	HILPDA	2.80	0.000409
ENSG00000186583	SPATC1	2.80	0.002485
ENSG00000121742	GJB6	2.79	0.00267
RP11-563J2.3	NA	2.79	0.047538
ENSG00000180871	CXCR2	2.78	3.43E-33
RP11-34P13.13	NA	2.78	0.040178
ENSG00000276404	MIR6835	2.77	0.000221
ENSG00000136689	IL1RN	2.77	4.20E-26
ENSG00000164542	KIAA0895	2.76	5.47E-05
ENSG00000112149	CD83	2.76	4.38E-30
ENSG00000277796	CCL3L3	2.74	0.005245
ENSG00000118997	DNAH7	2.73	0.037264
ENSG00000112715	VEGFA	2.73	5.22E-26
ENSG00000228978	TNF	2.73	7.91E-61
ENSG00000276219	ZNF676	2.72	0.028576
RP11-34P13.15	NA	2.72	5.94E-13
ENSG00000278567	CCL3	2.72	2.67E-12
ENSG00000198336	MYL4	2.72	0.005798
ENSG00000138587	MNS1	2.71	0.008969
ENSG00000147454	SLC25A37	2.70	1.20E-25
ENSG00000139890	REM2	2.70	2.15E-05
ENSG00000078401	EDN1	2.69	8.32E-08
ENSG00000139572	GPR84	2.68	3.03E-09
ENSG00000273793	DUSP8	2.68	5.16E-16
ENSG00000288183	MIR27A	2.66	0.002181
ENSG00000035664	DAPK2	2.63	3.40E-20
ENSG00000119121	TRPM6	2.62	4.01E-10
ENSG00000282508	LINC01002	2.62	9.37E-18
ENSG00000158615	PPP1R15B	2.62	1.60E-32
ENSG00000230342	FANCD2P2	2.61	0.047411
ENSG00000036672	USP2	2.59	0.023138
ENSG00000269220	LINC00528	2.59	0.025103
ENSG00000183762	KREMEN1	2.58	1.24E-12
TMEM56	NA	2.58	7.17E-10
RP11-20I23.2	NA	2.58	0.032824
ENSG00000165195	PIGA	2.58	8.82E-47
ENSG00000216819	TUBB2BP1	2.58	0.024786
ENSG00000283824	MIR22	2.58	4.84E-06
ENSG00000011422	PLAUR	2.57	3.66E-95
ENSG00000245532	NEAT1	2.57	8.22E-15
ENSG00000143507	DUSP10	2.56	3.57E-54
ENSG00000228278	ORM2	2.56	0.004403
ENSG00000131480	AOC2	2.56	3.62E-09
ENSG00000172216	CEBPB	2.55	1.89E-31
ENSG00000128594	LRRC4	2.55	2.94E-08
ENSG00000105656	ELL	2.55	8.59E-34
ENSG00000102554	KLF5	2.55	1.09E-15
ENSG00000069399	BCL3	2.55	8.31E-28
ENSG00000129038	LOXL1	2.55	3.98E-05
ENSG00000205710	C17orf107	2.55	3.65E-32
ENSG00000266618	MIR4742	2.55	0.004605
ENSG00000126803	HSPA2	2.53	8.97E-06
ENSG00000235527	HIPK1-AS1	2.53	0.042831
ENSG00000204160	ZDHHC18	2.53	5.14E-60
ENSG00000144802	NFKBIZ	2.52	5.88E-37
ENSG00000076864	RAP1GAP	2.52	0.004282
ENSG00000185897	FFAR3	2.49	9.61E-06
ENSG00000111837	MAK	2.49	3.72E-28
ENSG00000170956	CEACAM3	2.48	1.50E-16
ENSG00000145491	ROPN1L	2.47	0.000551
U91328.2	NA	2.46	2.53E-05
ENSG00000198355	PIM3	2.46	1.06E-16

ENSG00000173338	KCNK7	2.46	0.043328
ENSG00000148841	ITPRIP	2.45	2.42E-75
ENSG00000182541	LIMK2	2.44	2.73E-25
ENSG00000188536	HBA2	2.44	0.005199
ENSG00000063015	SEZ6	2.44	0.015362
ENSG00000173262	SLC2A14	2.43	3.58E-13
ENSG00000090104	RGS1	2.43	1.74E-20
ENSG00000131187	F12	2.43	1.07E-08
ENSG00000206172	HBA1	2.42	0.04827
RP11-452K12.6	NA	2.41	0.001709
RP11-96K19.2	NA	2.41	0.001121
RP11-305O6.4	NA	2.39	0.003478
U1	NA	2.38	0.019359
ENSG00000162783	IER5	2.38	2.93E-34
ENSG00000167470	MIDN	2.38	3.90E-21
ENSG00000288126	MIR24-2	2.37	0.001773
ENSG00000186105	LRRC70	2.37	1.37E-10
ENSG00000124882	EREG	2.37	0.00236
ENSG00000067082	KLF6	2.36	6.21E-38
C1ORF132	NA	2.35	6.85E-07
ENSG00000119508	NR4A3	2.34	5.42E-07
ENSG00000112137	PHACTR1	2.34	5.08E-20
ENSG00000258967	HMGN1P3	2.34	3.91E-09
ENSG00000281818	MANSC1	2.33	2.71E-08
AC074289.1	NA	2.33	0.020913
RP11-380G5.2	NA	2.32	5.60E-05
ENSG00000139289	PHLDA1	2.32	4.26E-09
ENSG00000230397	SPTLC1P1	2.32	4.80E-05
ENSG00000175352	NRIP3	2.31	0.030084
ENSG00000101460	MAP1LC3A	2.31	0.000321
AC068522.4	NA	2.31	0.001009
ENSG00000135083	CCNJL	2.30	7.89E-17
ENSG00000119801	YPEL5	2.29	1.21E-45
ENSG00000285027	ALG1L13P	2.29	2.01E-14
RP11-563J2.2	NA	2.28	0.029311
RP1-34B20.4	NA	2.27	5.01E-05
ENSG00000165030	NFIL3	2.27	1.12E-14
ENSG00000134201	GSTM5	2.27	0.003787
ENSG00000251022	THAP9-AS1	2.26	0.006152
ENSG00000138772	ANXA3	2.26	7.48E-07
ENSG00000187017	ESPN	2.25	0.028881
ENSG00000204345	CD300LD	2.25	0.026653
ENSG00000205189	ZBTB10	2.24	6.33E-51
ENSG00000144130	NT5DC4	2.24	0.006438
ENSG00000282800	MIR22HG	2.24	2.71E-13
ENSG00000110693	SOX6	2.24	0.000378
ENSG00000131669	NINJ1	2.24	1.67E-15
ENSG00000179820	MYADM	2.23	3.54E-36
ENSG00000244734	HBB	2.23	0.045807
ENSG00000163739	CXCL1	2.22	6.95E-06
CTD-3252C9.4	NA	2.22	0.001027
ENSG00000059804	SLC2A3	2.21	2.22E-16
ENSG00000110046	ATG2A	2.19	1.02E-30
ENSG00000203711	C6orf99	2.19	0.043766
ENSG00000222009	BTBD19	2.18	0.000223
ENSG00000185955	C7orf61	2.18	0.008908
ENSG00000174010	KLHL15	2.17	1.18E-31
ENSG00000115850	LCT	2.17	0.022621
ENSG00000206910	SNORA29	2.17	0.044988
ENSG00000132661	NXT1	2.17	1.44E-22
ENSG00000100644	HIF1A	2.17	2.90E-41
ENSG00000136244	IL6	2.17	0.000939
ENSG00000285069	SESN2	2.16	4.26E-15
ENSG00000178082	TWF1P1	2.16	0.007427
ENSG00000163660	CCNL1	2.16	6.15E-53
ENSG00000232804	HSPA1B	2.15	6.12E-16

ENSG00000113070	HBEGF	2.15	2.12E-06
ENSG00000163464	CXCR1	2.15	1.48E-08
ENSG00000112053	SLC26A8	2.15	7.27E-06
RP11-384B12.2	NA	2.14	0.047516
ENSG00000139112	GABARAPL1	2.14	2.95E-35
ENSG00000276858	FCAR	2.13	1.33E-13
ENSG00000102760	RGCC	2.13	1.91E-14
RP11-678G14.2	NA	2.13	0.010387
ENSG00000179094	PER1	2.13	5.11E-24
ENSG00000176597	B3GNT5	2.13	7.38E-10
ENSG00000140332	TLE3	2.12	7.04E-49
RP11-34P13.16	NA	2.12	4.35E-11
ENSG00000113916	BCL6	2.12	1.71E-23
ENSG00000188388	GOLGA6L3	2.11	0.005473
ENSG00000120129	DUSP1	2.11	3.86E-49
ENSG00000212304	SNORD12	2.11	6.68E-05
ENSG00000006652	IFRD1	2.11	4.66E-23
CHR22-38_28785274-29006793.1	NA	2.11	3.26E-09
ENSG00000274417	MIR6515	2.10	1.56E-11
ENSG00000104312	RIPK2	2.09	9.91E-20
ENSG00000093134	VNN3	2.09	1.23E-19
ENSG00000174871	CNIH2	2.09	0.000764
ENSG00000197279	ZNF165	2.08	0.000242
ENSG00000119535	CSF3R	2.08	3.60E-58
AC244230.1	NA	2.08	0.001074
ENSG00000125538	IL1B	2.08	3.15E-06
ENSG00000120156	TEK	2.07	0.041376
ENSG00000146232	NFKBIE	2.07	3.50E-12
ENSG00000284258	MIR8085	2.06	0.020614
ENSG00000196923	PDLIM7	2.06	2.19E-27
ENSG00000132470	ITGB4	2.05	0.001675
RP13-516M14.10	NA	2.05	2.27E-12
ENSG00000196668	LINC00173	2.05	0.001185
ENSG00000110848	CD69	2.04	1.44E-16
TTC25	NA	2.03	0.016787
ENSG00000235030	IER3	2.03	1.22E-16
ENSG00000167604	NFKBID	2.03	2.89E-18
ENSG00000176788	BASP1	2.03	3.43E-07
ENSG00000185201	IFITM2	2.03	3.06E-14
ENSG00000181409	AATK	2.03	1.37E-06
AC013394.2	NA	2.03	5.97E-06
ENSG00000059728	MXD1	2.02	1.29E-20
CASC1	NA	2.01	0.00172
ENSG00000157514	TSC22D3	2.01	2.21E-31
ENSG00000129244	ATP1B2	2.00	0.039793
ENSG00000128271	ADORA2A	2.00	5.20E-25
ENSG00000112245	PTP4A1	1.99	2.04E-24
ENSG00000124216	SNAI1	1.98	0.004268
ENSG00000120306	CYSTM1	1.98	0.000644
ENSG00000196083	IL1RAP	1.98	2.88E-28
ENSG00000116885	OSCP1	1.98	0.044979
ENSG00000197992	CLEC9A	1.97	0.002485
ENSG00000103257	SLC7A5	1.97	9.25E-17
ENSG00000260528	FAM157C	1.96	4.91E-07
ENSG00000170525	PFKFB3	1.96	3.66E-21
ENSG00000126524	SBDS	1.96	2.90E-41
ENSG00000155966	AFF2	1.95	3.16E-10
HIST1H1T	NA	1.95	0.042334
ENSG00000133101	CCNA1	1.94	0.003905
ENSG00000177410	ZFAS1	1.94	1.88E-08
ENSG00000112096	SOD2	1.92	6.20E-37
ENSG00000141096	DPEP3	1.92	9.04E-05
ENSG00000163661	PTX3	1.91	7.82E-05
RP3-437C15.1	NA	1.91	0.045432
ENSG00000160789	LMNA	1.90	3.55E-21
H1FX	NA	1.90	1.02E-14

ENSG00000182326	C1S	1.90	0.000187
ENSG00000118520	ARG1	1.89	1.63E-05
ENSG00000277402	MIR6891	1.89	1.97E-09
ENSG00000116337	AMPD2	1.89	1.86E-36
ENSG00000136826	KLF4	1.88	7.05E-10
ENSG00000151474	FRMD4A	1.88	0.010363
ENSG00000179833	SERTAD2	1.88	2.89E-44
ENSG00000197405	C5AR1	1.88	4.59E-18
ENSG00000167378	IRGQ	1.88	4.27E-10
ENSG00000288455	SEMA7A	1.87	1.34E-08
ENSG00000119922	IFIT2	1.87	4.18E-16
ENSG00000277443	MARCKS	1.86	5.25E-20
ENSG00000007944	MYLIP	1.85	3.79E-25
ENSG00000166900	STX3	1.85	1.71E-23
ENSG00000157388	CACNA1D	1.85	3.99E-05
ENSG00000258102	MAP1LC3B2	1.85	0.020131
ENSG00000103569	AQP9	1.85	9.65E-07
CH507-42P11.8	NA	1.84	0.001111
ENSG00000221420	SNORA81	1.84	1.27E-07
ENSG00000108179	PPIF	1.84	5.20E-49
AP000769.7	NA	1.84	0.034368
RP11-34P13.14	NA	1.84	2.74E-05
RP6-218J18.2	NA	1.84	0.000106
PVRL2	NA	1.83	0.004999
RP11-618G20.1	NA	1.83	4.27E-21
ENSG00000131015	ULBP2	1.83	0.034894
ENSG00000230724	LINC01001	1.83	1.47E-27
ENSG00000061656	SPAG4	1.83	0.026643
ENSG00000122644	ARL4A	1.83	2.95E-14
ENSG00000178053	MLF1	1.82	0.000127
ZNFX1-AS1_3	NA	1.82	0.040658
ENSG00000070495	JMJD6	1.82	1.07E-22
NEAT1_3	NA	1.82	0.015366
ENSG00000141232	TOB1	1.81	2.45E-23
ENSG00000198838	RYR3	1.81	0.009132
ENSG00000136541	ERMN	1.81	0.000193
ENSG00000177191	B3GNT8	1.81	0.000128
ENSG00000184588	PDE4B	1.81	1.26E-24
ENSG00000099985	OSM	1.81	1.07E-06
ENSG00000134107	BHLHE40	1.81	4.20E-30
ENSG00000137267	TUBB2A	1.80	8.30E-07
ENSG00000196358	NTNG2	1.80	9.91E-12
ENSG00000078804	TP53INP2	1.80	0.022395
ENSG00000143622	RIT1	1.79	1.77E-12
ENSG00000281484	BTG3	1.78	8.90E-09
ENSG00000042832	TG	1.77	2.24E-12
ENSG00000153234	NR4A2	1.76	3.15E-11
ENSG00000178381	ZFAND2A	1.76	8.77E-22
ENSG00000184205	TSPYL2	1.75	6.85E-15
ENSG00000013441	CLK1	1.75	2.18E-34
ENSG00000144597	EAF1	1.75	1.18E-17
ENSG00000160999	SH2B2	1.75	6.38E-14
ENSG00000070731	ST6GALNAC2	1.75	8.37E-05
ENSG00000105339	DENND3	1.75	4.25E-30
ENSG00000185972	CCIN	1.75	0.014609
ENSG00000132823	OSER1	1.74	1.25E-15
ENSG00000120709	FAM53C	1.74	6.48E-26
ENSG00000180953	ST20	1.73	5.39E-05
ENSG00000177374	HIC1	1.73	0.036651
ENSG00000143878	RHOB	1.73	2.08E-06
ENSG00000185262	UBALD2	1.72	4.41E-19
RP11-16E23.4	NA	1.71	1.81E-05
ENSG00000213714	FAM209B	1.70	0.020699
ENSG00000114315	HES1	1.70	0.032389
ENSG00000156232	WHAMM	1.69	1.22E-11
H3F3B	NA	1.68	1.73E-22

ENSG00000276761	CWC25	1.68	1.21E-33
ENSG00000264229	RNU4ATAC	1.68	0.000206
ENSG00000214193	SH3D21	1.68	2.99E-05
ENSG00000008438	PGLYRP1	1.68	1.89E-07
ENSG00000163659	TIPARP	1.68	5.86E-22
ENSG00000146592	CREB5	1.67	2.76E-12
ENSG00000178075	GRAMD1C	1.67	0.001061
ENSG00000269119	HNRNPA1P52	1.67	0.027859
ENSG00000129657	SEC14L1	1.67	1.70E-46
ENSG00000276615	KEL	1.67	0.03422
ENSG00000070761	CFAP20	1.66	6.52E-24
ENSG00000283060	ID3	1.65	0.000697
KIAA1324	NA	1.65	3.49E-05
ENSG00000242992	FTH1P4	1.65	0.022841
ENSG00000160051	IQCC	1.65	0.001702
RP11-756P10.3	NA	1.65	0.00404
ENSG00000166016	ABTB2	1.65	0.001176
ENSG00000138738	PRDM5	1.64	1.78E-05
ENSG00000232956	SNHG15	1.64	1.16E-06
ENSG00000132510	KDM6B	1.64	6.10E-27
ENSG00000169224	GCSAML	1.64	0.006437
ENSG00000281243	SNORD36B	1.64	0.039348
ENSG00000052749	RRP12	1.63	6.58E-16
ENSG00000124762	CDKN1A	1.63	1.39E-12
ENSG00000170345	FOS	1.63	3.52E-15
ENSG00000222365	SNORD12B	1.62	0.01436
RP11-548K23.11	NA	1.61	8.59E-19
SUV420H2	NA	1.61	0.000807
ENSG00000142178	SIK1	1.61	0.019174
ENSG00000080823	MOK	1.61	0.032741
ENSG00000054967	RELT	1.61	5.40E-17
ENSG00000209042	SNORD12C	1.61	0.001898
ENSG00000143751	SDE2	1.61	1.02E-24
ENSG00000135821	GLUL	1.61	3.77E-24
ENSG00000120051	CFAP58	1.61	0.004855
ENSG00000260727	SLC7A5P1	1.60	0.000438
ENSG00000169891	REPS2	1.60	9.65E-19
ENSG00000118689	FOXO3	1.60	8.77E-22
ENSG00000076053	RBM7	1.60	1.54E-12
ENSG00000155252	PI4K2A	1.59	2.55E-20
ENSG00000200530	SNORD35B	1.59	1.39E-05
C9ORF89	NA	1.59	4.76E-13
ENSG00000238317	SNORD11	1.59	0.003662
ENSG00000248966	BCLAF1P1	1.58	0.034368
ENSG00000067064	IDI1	1.58	1.36E-13
ENSG00000140941	MAP1LC3B	1.58	6.35E-11
ENSG00000138411	HECW2	1.58	0.001709
ENSG00000116016	EPAS1	1.57	6.18E-07
ENSG00000185950	IRS2	1.57	4.05E-12
ENSG00000116604	MEF2D	1.57	4.56E-14
RP5-1041C10.3	NA	1.57	0.003172
ENSG00000196296	ATP2A1	1.57	0.001655
ENSG00000143797	MBOAT2	1.55	1.26E-05
ENSG00000200463	SNORD118	1.55	0.000386
ENSG00000150991	UBC	1.54	1.61E-16
ENSG00000171988	JMJD1C	1.54	1.49E-33
ENSG00000111052	LIN7A	1.54	1.81E-12
ENSG00000115594	IL1R1	1.54	5.85E-05
ENSG00000105085	MED26	1.54	2.35E-06
ENSG00000168939	SPRY3	1.53	0.005045
ENSG00000119138	KLF9	1.53	4.28E-15
ENSG00000083812	ZNF324	1.53	4.61E-07
ENSG00000130066	SAT1	1.52	2.16E-10
CCRN4L	NA	1.52	0.001008
ENSG00000251441	RTEL1P1	1.52	0.007656
ENSG00000134815	DHX34	1.51	1.60E-18

ENSG00000108106	UBE2S	1.51	1.68E-09
ENSG00000133773	CCDC59	1.51	1.15E-24
ENSG00000163554	SPTA1	1.51	0.01993
RP11-473I1.9	NA	1.50	0.001852
ENSG00000127528	KLF2	1.50	4.27E-14
ENSG00000143384	MCL1	1.50	2.46E-16
ENSG00000151012	SLC7A11	1.50	0.027661
C10ORF131	NA	1.50	0.028784
ZNFX1-AS1_2	NA	1.49	0.010834
ENSG00000165175	MID1IP1	1.49	6.27E-13
ENSG00000112195	TREML2	1.49	7.50E-05
ENSG00000087903	RFX2	1.48	3.09E-08
ENSG00000123810	B9D2	1.48	0.002133
ENSG00000022567	SLC45A4	1.48	3.91E-09
ENSG00000165046	LETM2	1.48	5.15E-09
ENSG00000275990	NCF4	1.48	5.88E-26
ENSG00000196338	NLGN3	1.48	0.000274
RP11-299L17.1	NA	1.47	0.042968
ENSG00000118985	ELL2	1.47	4.29E-10
ENSG00000116514	RNF19B	1.47	5.09E-15
ENSG00000081041	CXCL2	1.47	0.015766
ENSG00000155090	KLF10	1.47	5.59E-08
ENSG00000285319	FTH1P2	1.47	8.64E-09
ENSG00000138069	RAB1A	1.46	9.50E-15
ENSG00000129355	CDKN2D	1.46	1.01E-08
FAM129A	NA	1.46	4.14E-08
ENSG00000173276	ZBTB21	1.46	3.68E-25
ENSG00000165650	PDZD8	1.46	2.78E-24
ENSG00000168209	DDIT4	1.46	3.18E-07
ENSG00000219085	NPM1P37	1.46	0.011615
RP11-810P12.7	NA	1.45	0.003492
ENSG00000202538	RNU4-2	1.45	0.000993
ENSG00000167173	C15orf39	1.45	1.12E-09
ENSG00000105855	ITGB8	1.45	0.041975
ENSG00000134531	EMP1	1.44	0.004801
ENSG00000155330	C16orf87	1.44	3.58E-13
ENSG00000120705	ETF1	1.44	7.59E-15
RP11-212D19.4	NA	1.44	3.45E-09
ENSG00000091536	MYO15A	1.44	0.024287
ENSG00000109674	NEIL3	1.44	5.78E-09
ENSG00000076604	TRAF4	1.44	0.000963
ENSG00000198400	NTRK1	1.43	0.00396
ENSG00000137449	CPEB2	1.43	3.44E-18
ENSG00000114270	COL7A1	1.43	0.000963
ENSG00000278119	GOLGA8J	1.43	0.002376
ENSG00000113734	BNIP1	1.43	5.85E-07
ENSG00000182487	NCF1B	1.42	3.49E-10
ENSG00000117000	RLF	1.42	5.19E-24
ENSG00000188026	RILPL1	1.42	0.000244
ENSG00000141582	CBX4	1.42	1.66E-12
ENSG00000225553	PHF1	1.42	1.29E-08
ENSG00000242227	HLA-L	1.42	0.027706
ENSG00000069956	MAPK6	1.41	4.36E-09
ENSG00000146278	PNRC1	1.41	2.63E-39
ENSG00000168264	IRF2BP2	1.41	1.23E-18
ENSG00000230204	FTH1P5	1.41	0.001038
ENSG00000147459	DOCK5	1.41	1.95E-11
ENSG00000104973	MED25	1.41	2.27E-12
ENSG00000206680	SNORD21	1.41	0.011389
FAM46C	NA	1.41	1.38E-06
RP11-73E17.2	NA	1.41	0.042199
ENSG00000128590	DNAJB9	1.40	2.82E-10
ENSG00000119950	MXI1	1.40	1.02E-19
ENSG00000135241	PNPLA8	1.40	2.56E-14
ENSG00000275043	SNORD25	1.40	0.041443
RP11-863K10.4	NA	1.40	0.033833

ENSG00000125812	GZF1	1.40	1.32E-07
ENSG00000237264	FTH1P11	1.40	1.07E-09
ENSG00000163545	NUAK2	1.39	8.71E-07
GRASP	NA	1.39	4.01E-06
ENSG00000109519	GRPEL1	1.38	8.52E-08
ENSG00000156860	FBRS	1.38	6.55E-15
HIST2H2BB	NA	1.38	0.004027
ENSG00000167996	FTH1	1.38	1.24E-14
ENSG00000077150	NFKB2	1.38	7.05E-10
ENSG00000171223	JUNB	1.38	1.83E-11
ENSG00000233013	FAM157B	1.38	3.73E-06
ENSG00000169895	SYAP1	1.37	1.01E-12
ENSG00000138621	PPCDC	1.37	4.39E-16
RP11-157G21.2	NA	1.37	0.006498
ENSG00000116991	SIPA1L2	1.37	0.00193
ENSG00000242960	FTH1P23	1.37	3.08E-08
ENSG00000125898	FAM110A	1.37	0.000233
ENSG00000122862	SRGN	1.37	9.41E-13
ENSG00000073756	PTGS2	1.37	0.000301
ENSG00000197063	MAFG	1.36	0.033046
ENSG00000166947	EPB42	1.36	0.049949
ENSG00000185650	ZFP36L1	1.36	3.44E-10
ENSG00000160908	ZNF394	1.36	2.78E-24
ENSG00000165997	ARL5B	1.36	2.87E-06
ENSG00000219507	FTH1P8	1.36	1.55E-11
ENSG00000232187	FTH1P7	1.36	1.13E-07
ENSG00000138166	DUSP5	1.35	8.04E-06
ENSG00000145632	PLK2	1.35	0.000194
ENSG00000147872	PLIN2	1.35	4.32E-06
ENSG00000126861	OMG	1.35	0.015271
ENSG00000198833	UBE2J1	1.35	2.50E-08
ENSG00000145335	SNCA	1.34	0.001038
ENSG00000064547	LPAR2	1.34	9.65E-07
ENSG00000162702	ZNF281	1.34	7.14E-25
ENSG00000075426	FOSL2	1.34	1.69E-10
ENSG00000288219	TNNI2	1.34	5.60E-06
ENSG00000162910	MRPL55	1.34	0.013043
ENSG00000148200	NR6A1	1.33	0.00305
ENSG00000107338	SHB	1.33	0.032111
ENSG00000029363	BCLAF1	1.33	1.35E-28
ENSG00000182871	COL18A1	1.33	0.000657
ENSG00000137494	ANKRD42	1.33	3.40E-05
ENSG00000205755	CRLF2	1.33	0.039971
ENSG00000100284	TOM1	1.32	1.99E-20
ENSG00000079385	CEACAM1	1.32	0.000194
ENSG00000102096	PIM2	1.32	2.72E-07
ENSG00000095574	IKZF5	1.32	9.48E-13
ENSG00000122477	LRRC39	1.32	0.027845
ENSG00000226564	FTH1P20	1.31	1.64E-13
RP5-998N21.10	NA	1.31	0.007929
ENSG00000100368	CSF2RB	1.31	5.13E-11
ENSG00000109756	RAPGEF2	1.30	6.16E-16
ENSG00000102119	EMD	1.30	9.56E-32
ENSG00000158480	SPATA2	1.30	5.78E-05
ENSG00000154269	ENPP3	1.30	0.0228
ENSG00000057704	TMCC3	1.30	7.08E-09
ENSG00000137414	FAM8A1	1.30	2.04E-10
ENSG00000112303	VNN2	1.30	0.001531
ENSG00000151726	ACSL1	1.30	4.97E-07
ENSG00000225648	SBDSP1	1.30	3.90E-14
ENSG00000152409	JMY	1.30	1.54E-07
ENSG00000165115	KIF27	1.29	2.80E-06
ENSG00000225022	UBE2D3P1	1.29	0.006943
RP11-582J16.5	NA	1.29	9.74E-07
ENSG00000244115	DNAJC25-GNG10	1.29	0.000322
ENSG00000242616	GNG10	1.29	0.000477

ENSG00000176289	IDSP1	1.29	0.00666
ENSG00000143862	ARL8A	1.29	9.53E-08
ENSG00000135636	DYSF	1.29	0.000208
TWISTNB	NA	1.29	7.45E-11
SELK	NA	1.29	7.16E-12
ENSG00000162616	DNAJB4	1.29	3.36E-06
ENSG00000059769	DNAJC25	1.29	0.000177
ENSG00000089818	NECAP1	1.29	5.51E-20
ENSG00000167565	SERTAD3	1.29	8.84E-05
ENSG00000164056	SPRY1	1.29	0.004083
RP11-107E5.2	NA	1.29	2.66E-05
EMR3	NA	1.28	2.84E-10
ENSG00000179335	CLK3	1.27	4.24E-24
ENSG00000101236	RNF24	1.27	1.45E-06
ENSG00000130821	SLC6A8	1.27	0.005657
ENSG00000136527	TRA2B	1.27	7.19E-31
ENSG00000196428	TSC22D2	1.27	3.52E-17
ABBA01017803.1	NA	1.27	5.06E-05
ENSG00000132002	DNAJB1	1.27	3.73E-20
ENSG00000100226	GTPBP1	1.26	2.36E-18
ENSG00000128512	DOCK4	1.26	0.001615
ENSG00000125347	IRF1	1.26	1.02E-10
ENSG00000116752	BCAS2	1.26	9.73E-14
RP11-44F14.1	NA	1.26	3.76E-05
ENSG00000169902	TPST1	1.26	0.00154
ENSG00000129295	LRRC6	1.26	1.23E-06
ENSG00000198858	R3HDM4	1.26	6.27E-10
ENSG00000207741	MIR590	1.25	0.036675
ENSG00000143367	TUFT1	1.25	0.001309
ENSG00000196371	FUT4	1.25	0.000144
ENSG00000122547	EEPD1	1.24	9.92E-10
ENSG00000164691	TAGAP	1.24	2.37E-22
ENSG00000138433	CIR1	1.24	1.07E-19
FAM212B	NA	1.24	0.003696
ENSG00000249850	KRT18P31	1.24	0.007831
ENSG00000163751	CPA3	1.24	0.017711
SETD8	NA	1.24	2.75E-08
ENSG00000263776	SNORA4	1.23	0.000188
H1F0	NA	1.23	0.013902
ENSG00000288512	C1R	1.23	0.001984
ENSG00000102225	CDK16	1.23	1.55E-12
ENSG00000163171	CDC42EP3	1.23	6.57E-05
ENSG00000238942	SNORD2	1.23	0.000286
ENSG00000134758	RNF138	1.23	2.39E-09
ENSG00000177173	NAP1L4P1	1.23	0.031054
ENSG00000156313	RPGR	1.23	3.93E-11
ENSG00000104450	SPAG1	1.23	4.19E-05
ENSG00000234743	EIF5AP4	1.23	0.000367
HIST1H2AK	NA	1.23	3.74E-06
ENSG00000104856	RELB	1.23	5.20E-09
ENSG00000154710	RABGEF1	1.23	6.65E-10
HIST2H2BF	NA	1.22	3.21E-05
ENSG00000227376	FTH1P16	1.22	2.28E-06
C20ORF24	NA	1.22	1.08E-06
ENSG00000223361	FTH1P10	1.22	2.11E-12
ENSG00000213462	ERV3-1	1.22	6.66E-06
ENSG00000276336	SCARF1	1.21	0.000738
ENSG00000116273	PHF13	1.21	0.000286
ENSG00000121671	CRY2	1.21	0.000141
DFNB31	NA	1.21	0.006318
ENSG00000265185	SNORD3B-1	1.20	0.008533
ENSG00000166839	ANKDD1A	1.20	0.004582
TGIF2-C20ORF24	NA	1.20	2.67E-06
ENSG00000129204	USP6	1.20	0.039247
ENSG00000277025	MBOAT7	1.20	1.90E-06
ENSG00000165355	FBXO33	1.20	9.98E-19

ENSG00000284360	MIR6125	1.20	4.54E-05
RP11-25K21.6	NA	1.20	1.11E-10
ENSG00000033327	GAB2	1.20	6.34E-12
ENSG00000186642	PDE2A	1.20	0.035287
AP000347.2	NA	1.20	3.32E-05
ENSG00000282905	NFKBIB	1.19	0.001396
ENSG00000113742	CPEB4	1.19	8.19E-10
ENSG00000285343	PPP1R3B	1.19	0.000178
ENSG00000170779	CDCA4	1.18	0.035905
ENSG00000156671	SAMD8	1.18	1.29E-11
ENSG00000162231	NXF1	1.17	2.28E-15
ENSG00000166579	NDEL1	1.17	3.52E-12
ENSG00000114120	SLC25A36	1.17	4.74E-12
ENSG00000129315	CCNT1	1.17	2.75E-22
ENSG00000168389	MFSD2A	1.17	1.37E-08
ENSG00000180667	YOD1	1.17	1.55E-12
ENSG00000130844	ZNF331	1.17	7.72E-06
ENSG00000127666	TICAM1	1.17	2.37E-07
ENSG00000110446	SLC15A3	1.16	1.71E-12
ENSG00000164938	TP53INP1	1.16	1.39E-11
ENSG00000165178	NCF1C	1.16	0.001472
ENSG00000107968	MAP3K8	1.16	2.27E-14
ENSG00000139832	RAB20	1.16	0.004984
ENSG00000185215	TNFAIP2	1.16	6.60E-09
HIST1H1C	NA	1.16	2.62E-07
ENSG00000179869	ABCA13	1.16	0.026353
ENSG00000161921	CXCL16	1.15	0.00059
ENSG00000166987	MBD6	1.15	2.87E-15
HIST1H1E	NA	1.15	1.07E-06
KIAA1683	NA	1.15	0.020046
ENSG00000115956	PLEK	1.15	1.08E-06
ENSG00000123700	KCNJ2	1.15	0.000306
ENSG00000154813	DPH3	1.15	2.62E-06
ENSG00000179750	APOBEC3B	1.15	3.55E-05
RP11-599B13.6	NA	1.15	1.02E-09
ENSG00000177169	ULK1	1.15	4.26E-09
ENSG00000143226	FCGR2A	1.15	2.10E-10
ENSG00000131051	RBM39	1.14	2.97E-28
ENSG00000164463	CREBRF	1.14	8.61E-15
ENSG00000165312	OTUD1	1.13	2.95E-12
RP11-274B21.1	NA	1.13	8.69E-08
ENSG00000239305	RNF103	1.13	1.45E-11
ENSG00000109332	UBE2D3	1.13	4.23E-11
ENSG00000106245	BUD31	1.13	3.03E-09
ENSG00000197780	TAF13	1.13	9.49E-07
ENSG00000196850	PPTC7	1.13	3.67E-12
ENSG00000258186	SLC7A5P2	1.12	0.005591
ENSG00000162066	AMDHD2	1.12	4.44E-07
ENSG00000262156	APOBEC3A	1.12	4.32E-06
ENSG00000135604	STX11	1.12	4.07E-08
ENSG00000136026	CKAP4	1.12	7.73E-10
ENSG00000240433	LY6G5B	1.11	5.78E-08
AC027612.1	NA	1.11	0.02502
ENSG00000170340	B3GNT2	1.10	0.001027
ENSG00000163376	KBTBD8	1.10	1.92E-06
ENSG00000090061	CCNK	1.10	3.74E-14
ENSG00000185261	KIAA0825	1.10	0.000838
ENSG00000065665	SEC61A2	1.10	6.63E-05
ENSG00000151948	GLT1D1	1.10	7.21E-06
ENSG00000275911	NDE1	1.10	2.71E-15
ENSG00000235307	BRD2	1.10	3.59E-15
ENSG00000213362	FTH1P12	1.10	5.81E-06
ENSG00000136929	HEMGN	1.10	0.016855
ENSG00000151239	TWF1	1.10	2.20E-11
ENSG00000132141	CCT6B	1.10	0.037235
ENSG00000188229	TUBB4B	1.10	4.76E-18

ENSG00000101782	RIOK3	1.09	4.73E-08
ENSG00000187627	RGPD1	1.09	0.049949
ENSG00000101665	SMAD7	1.09	0.000127
HIAT1	NA	1.08	2.91E-13
ENSG00000086288	NME8	1.08	1.77E-05
ENSG00000072401	UBE2D1	1.08	0.001994
ENSG00000101493	ZNF516	1.08	4.73E-12
ENSG00000172409	CLP1	1.08	5.14E-05
ENSG00000220205	VAMP2	1.08	3.92E-10
ENSG00000274131	CEACAM4	1.08	0.00052
ENSG00000119048	UBE2B	1.07	9.34E-07
ENSG00000145819	ARHGAP26	1.07	1.20E-14
ENSG00000196862	RGPD4	1.07	0.006199
ENSG00000051108	HERPUD1	1.07	7.01E-08
ENSG00000171310	CHST11	1.07	2.36E-10
ENSG00000152926	ZNF117	1.07	1.94E-09
ENSG00000276788	SNORD26	1.07	0.00112
ENSG00000102543	CDADC1	1.07	4.14E-07
ENSG00000113369	ARRDC3	1.06	7.73E-08
ENSG00000168556	ING2	1.06	4.83E-07
ENSG00000243335	KCTD7	1.06	6.66E-08
ENSG00000105993	DNAJB6	1.06	2.93E-10
ENSG00000169180	XPO6	1.05	4.52E-24
ENSG00000034152	MAP2K3	1.05	1.31E-13
ENSG00000115520	COQ10B	1.05	9.94E-11
ENSG00000120063	GNA13	1.05	2.67E-15
ENSG00000173812	EIF1	1.05	1.47E-25
ENSG00000255423	EBLN2	1.05	0.000963
ENSG00000168066	SF1	1.05	4.43E-21
ENSG00000135114	OASL	1.05	0.007521
ENSG00000184602	SNN	1.05	0.001982
ENSG00000169155	ZBTB43	1.05	3.26E-05
ENSG00000132819	RBM38	1.04	2.47E-05
ENSG00000123636	BAZ2B	1.04	4.43E-10
ENSG00000179029	TMEM107	1.04	0.004377
ENSG00000241852	C8orf58	1.04	0.003798
ENSG00000172059	KLF11	1.04	5.87E-07
H3F3C	NA	1.04	0.044126
ENSG00000204455	TRIM51BP	1.04	0.012004
ENSG00000007516	BAIAP3	1.04	0.042525
ENSG00000285360	LONRF1	1.04	1.21E-06
ENSG00000114784	EIF1B	1.04	3.43E-07
ENSG00000085117	CD82	1.04	1.21E-08
ENSG00000179119	SPTY2D1	1.04	4.97E-13
ENSG00000117115	PADI2	1.03	1.63E-06
ENSG00000105722	ERF	1.03	3.67E-05
ENSG00000135404	CD63	1.03	0.00054
ENSG00000162664	ZNF326	1.03	4.11E-09
ENSG00000125772	GPCPD1	1.03	6.11E-17
ENSG00000145780	FEM1C	1.03	1.71E-08
ENSG00000134686	PHC2	1.03	1.92E-08
ENSG00000164663	USP49	1.03	0.005781
ENSG00000166889	PATL1	1.03	3.31E-14
ENSG00000101596	SMCHD1	1.03	2.50E-16
ENSG00000272533	SNORA28	1.03	0.004951
ENSG00000068323	TFE3	1.02	9.31E-09
ENSG00000160570	DEDD2	1.02	5.16E-05
ENSG00000161526	SAP30BP	1.02	1.66E-05
AC069368.3	NA	1.02	0.000108
ENSG00000118515	SGK1	1.02	0.000475
ENSG00000150977	RILPL2	1.02	2.58E-07
ENSG00000215301	DDX3X	1.02	1.17E-07
ENSG00000130164	LDLR	1.02	0.002139
ENSG00000275272	YTHDC1	1.01	6.83E-14
ENSG00000235754	APOM	1.01	0.021798
ENSG00000196663	TECPR2	1.01	5.02E-17

ENSG00000230701	FBXW4P1	1.01	0.007187
RP11-676M6.1	NA	1.01	0.000745
ENSG00000198901	PRC1	1.01	0.002696
ENSG00000274166	GOLGA6L10	1.00	0.031238
ENSG00000184305	CCSER1	1.00	0.012813
ENSG00000127954	STEAP4	1.00	0.001522
ATP6C	NA	1.00	0.001447
ENSG00000164934	DCAF13	-1.00	0.000144
ENSG00000031691	CENPQ	-1.00	0.007518
FTSJ2	NA	-1.00	5.96E-05
ENSG00000185085	INTS5	-1.00	3.70E-06
ENSG00000114491	UMPS	-1.00	1.03E-12
ENSG00000166750	SLFN5	-1.01	8.16E-07
AARS	NA	-1.01	1.41E-20
ENSG00000123870	ZNF137P	-1.01	0.002464
ENSG00000131943	C19orf12	-1.01	9.26E-08
ENSG00000196466	ZNF799	-1.01	1.73E-05
ENSG00000174946	GPR171	-1.01	0.001985
ENSG00000185220	PGBD2	-1.02	3.11E-05
ENSG00000174123	TLR10	-1.02	0.029427
ENSG00000117450	PRDX1	-1.02	0.00082
ENSG00000138829	FBN2	-1.02	2.52E-07
ENSG00000147124	ZNF41	-1.02	2.71E-09
ENSG00000124374	PAIP2B	-1.02	0.039221
ENSG00000186376	ZNF75D	-1.03	2.67E-06
ENSG00000106351	AGFG2	-1.03	1.80E-08
ENSG00000189144	ZNF573	-1.03	0.002379
ENSG00000270011	ZNF559-ZNF177	-1.03	4.60E-05
ENSG00000213793	ZNF888	-1.03	0.000142
ENSG00000171574	ZNF584	-1.03	0.037012
ENSG00000197044	ZNF441	-1.04	2.71E-05
ENSG00000132623	ANKEF1	-1.04	0.006957
ENSG00000132313	MRPL35	-1.04	0.001055
ENSG00000212916	MAP10	-1.05	0.004282
ENSG00000262473	GART	-1.05	6.11E-12
ENSG00000130347	RTN4IP1	-1.05	0.002886
ENSG00000181004	BBS12	-1.05	0.008825
ENSG00000167977	KCTD5	-1.05	1.93E-06
ENSG00000153767	GTF2E1	-1.05	4.62E-07
ENSG00000178537	SLC25A20	-1.06	3.87E-05
ENSG00000166477	LEO1	-1.06	1.79E-09
ENSG00000117528	ABCD3	-1.06	3.39E-09
ENSG00000177971	IMP3	-1.06	0.008573
ENSG00000117010	ZNF684	-1.06	0.027313
ENSG00000185875	THNSL1	-1.06	0.009345
FAM129B	NA	-1.06	0.003514
ENSG00000263002	ZNF234	-1.07	4.52E-09
ENSG00000131115	ZNF227	-1.07	1.01E-08
ENSG00000146833	TRIM4	-1.07	1.52E-09
ENSG00000167384	ZNF180	-1.07	1.12E-07
BAIAP2-AS1	NA	-1.08	0.032472
ENSG00000172171	TEFM	-1.08	0.000444
ENSG00000164168	TMEM184C	-1.08	1.04E-06
ENSG00000281526	ZNF546	-1.08	0.000438
ENSG00000120053	GOT1	-1.08	3.12E-05
ENSG00000243414	TICAM2	-1.08	0.031086
ENSG00000180257	ZNF816	-1.08	8.93E-08
ENSG00000105136	ZNF419	-1.09	3.94E-05
ENSG00000165617	DACT1	-1.09	0.000374
ENSG00000181610	MRPS23	-1.09	0.000153
ENSG00000139193	CD27	-1.09	0.001319
ENSG00000211794	TRAV12-3	-1.09	0.027324
ENSG00000171860	C3AR1	-1.10	0.018741
ENSG00000197362	ZNF786	-1.10	0.000161
ENSG00000196290	NIF3L1	-1.10	9.03E-07
ENSG00000284816	EPHA1	-1.10	0.046456

ENSG00000117322	CR2	-1.10	0.029041
ENSG00000178966	RMI1	-1.10	0.003842
ENSG00000234284	ZNF879	-1.10	0.004942
ENSG00000112118	MCM3	-1.10	2.10E-13
ENSG00000185869	ZNF829	-1.11	0.006285
ENSG00000167637	ZNF283	-1.11	0.000174
ENSG00000211662	IGLV3-21	-1.11	0.013068
ENSG00000065183	WDR3	-1.11	1.45E-20
ENSG00000259040	BLOC1S5-TXNDC5	-1.11	2.41E-13
ENSG00000167635	ZNF146	-1.11	6.19E-08
ENSG00000215271	HOMEZ	-1.11	0.00172
ENSG00000242852	ZNF709	-1.11	0.000522
DHFRL1	NA	-1.11	2.71E-05
C2ORF44	NA	-1.12	0.000234
ENSG00000168061	SAC3D1	-1.12	0.033047
ENSG00000141040	ZNF287	-1.12	0.003852
ENSG00000167380	ZNF226	-1.12	7.55E-11
ENSG00000274854	IGLV1-44	-1.12	0.001102
ENSG00000178988	MRFAP1L1	-1.12	2.14E-06
ENSG00000161298	ZNF382	-1.12	0.002361
ENSG00000081692	JMJD4	-1.13	0.020792
ENSG00000100721	TCL1A	-1.13	0.010746
ENSG00000196329	GIMAP5	-1.13	4.78E-07
ENSG00000163508	EOMES	-1.13	0.009047
C10ORF2	NA	-1.13	0.000452
ENSG00000107789	MINPP1	-1.13	0.000432
ENSG00000140987	ZSCAN32	-1.14	0.000145
ENSG00000145740	SLC30A5	-1.14	2.41E-13
ENSG00000135446	CDK4	-1.14	8.58E-11
ENSG00000154734	ADAMTS1	-1.14	0.003477
ENSG00000174327	SLC16A13	-1.15	0.00283
ENSG00000186230	ZNF749	-1.15	0.006175
SGK223	NA	-1.15	0.00013
ENSG00000126353	CCR7	-1.15	0.007258
ENSG00000033011	ALG1	-1.15	1.00E-14
ENSG00000185670	ZBTB3	-1.16	0.002077
ENSG00000189369	GSPT2	-1.16	7.22E-06
ENSG00000100413	POLR3H	-1.16	9.28E-06
ENSG00000254004	ZNF260	-1.17	5.31E-06
ENSG00000277016	IGHG4	-1.17	1.42E-06
ENSG00000186666	BCDIN3D	-1.17	0.026143
ENSG00000198298	ZNF485	-1.17	0.010146
ENSG00000198182	ZNF607	-1.18	0.000157
ENSG00000100711	ZFYVE21	-1.18	0.010825
ENSG00000132357	CARD6	-1.18	0.000483
ENSG00000211801	TRAV21	-1.19	0.005591
ENSG00000172315	TP53RK	-1.19	2.15E-05
ENSG00000188321	ZNF559	-1.19	2.80E-14
ENSG00000138061	CYP1B1	-1.19	0.00855
ENSG00000186272	ZNF17	-1.19	1.36E-10
ENSG00000116990	MYCL	-1.19	0.005885
ENSG00000131849	ZNF132	-1.19	0.003142
ENSG00000284944	MED18	-1.21	0.000268
METTL13	NA	-1.21	1.53E-15
HN1L	NA	-1.21	0.000123
C12ORF49	NA	-1.21	0.00102
ENSG00000160791	CCR5	-1.22	0.000993
CTD-2528L19.4	NA	-1.22	0.042883
ENSG00000083817	ZNF416	-1.22	1.68E-05
ENSG00000132423	COQ3	-1.22	0.007141
ENSG00000082516	GEMIN5	-1.23	8.00E-19
ENSG00000111875	ASF1A	-1.24	2.86E-12
AP001372.2	NA	-1.24	0.017543
ENSG00000197933	ZNF823	-1.24	2.85E-08
ENSG00000211790	TRAV8-4	-1.24	6.69E-06
ENSG00000197863	ZNF790	-1.24	0.010093

ENSG00000256294	ZNF225	-1.24	2.75E-08
ENSG00000229186	ADAM1A	-1.24	0.000552
ENSG00000136014	USP44	-1.24	0.003869
ENSG00000133116	KL	-1.24	0.029794
ENSG00000284049	MIR650	-1.25	0.005401
RP11-97O12.7	NA	-1.25	3.31E-06
ENSG00000175691	ZNF77	-1.25	0.001757
ENSG00000133574	GIMAP4	-1.26	6.80E-05
ENSG00000105088	OLFM2	-1.27	0.013458
ENSG00000113552	GNPDA1	-1.27	5.56E-07
ENSG00000211815	TRAV36DV7	-1.27	0.031674
ENSG00000132801	ZSWIM3	-1.27	0.013068
ENSG00000131203	IDO1	-1.27	0.022545
RP11-1094M14.7	NA	-1.27	0.002043
ENSG00000184432	COPB2	-1.27	8.23E-08
ENSG00000255561	FDXACB1	-1.28	0.000598
ENSG00000211648	IGLV1-47	-1.28	1.81E-06
ENSG00000179933	C14orf119	-1.29	2.22E-05
ENSG00000282436	TRBV11-2	-1.29	0.023698
ENSG00000171806	METTL18	-1.29	0.002518
ENSG00000275111	ZNF2	-1.29	0.000705
ENSG00000176024	ZNF613	-1.29	0.002577
HIATL1	NA	-1.29	6.37E-08
ENSG00000091106	NLRC4	-1.29	7.29E-11
ENSG00000171428	NAT1	-1.30	0.005657
ENSG00000239264	TXNDC5	-1.31	2.14E-15
ENSG00000211793	TRAV9-2	-1.31	0.000711
ENSG00000181007	ZFP82	-1.31	0.001355
ENSG00000197128	ZNF772	-1.31	0.001379
ENSG00000141378	PTRH2	-1.32	1.02E-05
ENSG00000197841	ZNF181	-1.32	6.59E-05
ENSG00000132763	MMACHC	-1.32	0.000118
ENSG00000259494	MRPL46	-1.32	3.72E-05
ENSG00000211799	TRAV19	-1.33	0.000661
ENSG00000120784	ZFP30	-1.34	4.88E-07
ENSG00000168329	CX3CR1	-1.35	0.000717
ENSG00000116771	AGMAT	-1.35	0.001271
ENSG00000277071	NLRP7	-1.35	0.034848
ENSG00000165118	C9orf64	-1.38	8.69E-05
ENSG00000187626	ZKSCAN4	-1.38	7.33E-05
ENSG00000221909	FAM200A	-1.39	0.004997
ENSG00000163686	ABHD6	-1.39	3.54E-05
ENSG00000211789	TRAV12-2	-1.42	0.014243
ENSG00000211779	TRAV5	-1.43	0.029892
ENSG00000274349	ZNF658	-1.43	1.88E-06
ENSG00000165672	PRDX3	-1.44	7.81E-08
ENSG00000281894	ZKSCAN7	-1.45	0.000686
ENSG00000148468	FAM171A1	-1.47	0.040677
ENSG00000179144	GIMAP7	-1.47	2.45E-09
ENSG00000211806	TRAV25	-1.47	0.04887
ENSG00000137078	SIT1	-1.47	0.000243
ENSG00000170915	PAQR8	-1.50	1.17E-06
ENSG00000204611	ZNF616	-1.50	4.56E-18
ENSG00000188610	FAM72B	-1.52	0.009443
ENSG00000281970	TRBV6-1	-1.52	0.000666
ENSG00000167554	ZNF610	-1.52	0.008518
ENSG00000133561	GIMAP6	-1.52	1.91E-29
ENSG00000282466	TRBV5-4	-1.54	0.001265
ENSG00000136514	RTP4	-1.55	0.006329
ENSG00000256553	TRAV1-2	-1.56	0.005368
ENSG00000180917	CMTR2	-1.57	9.14E-13
ENSG00000121807	CCR2	-1.57	4.61E-11
ENSG00000281661	ZNF501	-1.57	0.001837
ENSG00000211809	TRAV27	-1.57	0.044707
ENSG00000131634	TMEM204	-1.57	0.032818
ENSG00000171115	GIMAP8	-1.58	2.73E-13

ENSG00000104635	SLC39A14	-1.60	0.002258
ENSG00000137168	PPIL1	-1.61	5.93E-08
ENSG00000197050	ZNF420	-1.63	4.40E-08
ENSG00000117016	RIMS3	-1.70	0.004242
ENSG00000134504	KCTD1	-1.70	0.0267
ENSG00000077152	UBE2T	-1.79	0.006285
ENSG00000176222	ZNF404	-1.82	0.00949
ENSG00000183647	ZNF530	-1.84	1.05E-12
ENSG00000173114	LRRN3	-1.85	0.012191
ENSG00000267508	ZNF285	-1.90	0.000178
ENSG00000198342	ZNF442	-1.97	1.10E-05
ENSG00000127903	ZNF835	-2.04	0.01675
ENSG00000274600	RIMBP3B	-2.05	0.011917
ENSG00000211658	IGLV3-27	-2.07	0.007789
RP11-932O9.8	NA	-2.10	0.023755
ENSG00000122877	EGR2	-2.11	4.13E-11
ENSG00000130037	KCNA5	-2.22	0.030632
ENSG00000244693	CTAGE8	-2.23	0.023797
ENSG00000281944	IGHV3-6	-2.57	0.038374
ENSG00000000003	TSPAN6	-2.61	0.02321
ENSG00000172215	CXCR6	-2.69	0.000197
ENSG00000211623	IGKV2D-26	-4.47	0.013706
ENSG00000226212	TRGV6	-5.92	0.031841
ENSG00000285251	DEFA5	-7.94	5.37E-09
