# Supplementary material for: Peripheral blood mononuclear cell phenotype and function are maintained after overnight shipping of whole blood
Source: Sci Rep. 2022 Nov 19;12:19920. doi: 10.1038/s41598-022-24550-6 (PMC9675784; doi:10.1038/s41598-022-24550-6)
Supplement: Supplementary file 4 — Supplementary Information 4. [file 41598_2022_24550_MOESM4_ESM.docx]

*Supplemental Figure 1. PBMC RNAseq QC and genes differentially expressed after overnight shipping.*

(A) Distribution of normalized and transformed FPKM values for triplicate samples from fresh blood (X0h) and blood shipped overnight (X24h). (B) Volcano plot showing genes differentially upregulated (red) or downregulated (blue) using a maximum FDR of 0.05 and a minimum 2-fold change in expression between groups.
